# Dual pancreatic carcinomas: clonally related or independent primaries?

**DOI:** 10.1038/s41698-026-01313-4

**Published:** 2026-02-08

**Authors:** Joshua D. Schoenfeld, Vignesh Ravichandran, Zeynep Tarcan, Hulya Sahin-Ozkan, Allison L. Richards, Nadeem Bilani, Joanne F. Chou, Catherine A. O’Connor, David McManamon, Anna M. Varghese, Fergus Keane, Michael I. D’Angelica, William R. Jarnagin, Jeffrey Drebin, Diane Reidy-Lagunes, Alice C. Wei, Sree B. Chalasani, Fiyinfolu Balogun, Wungki Park, Kenneth H. Yu, Kevin Soares, Mark T. A. Donoghue, Christine Iacobuzio-Donahue, Zsofia K. Stadler, Marinela Capanu, Olca Basturk, Eileen M. O’Reilly

**Affiliations:** 1 Department of Medicine, Memorial Sloan Kettering Cancer Center, New York, NY USA; 2 David M. Rubenstein Center for Pancreatic Cancer Research, Memorial Sloan Kettering Cancer Center, New York, NY USA; 3 Center for Molecular Oncology, Memorial Sloan Kettering Cancer Center, New York, NY USA; 4 Department of Pathology, Memorial Sloan Kettering Cancer Center, New York, NY USA; 5 Department of Epidemiology and Biostatistics, Memorial Sloan Kettering Cancer Center, New York, NY USA; 6 Department of Surgery, Memorial Sloan Kettering Cancer Center, New York, NY USA

**Keywords:** Cancer, Oncology

## Abstract

Dual pancreatic cancers, either synchronous or metachronous in presentation, are a rare occurrence. It remains unclear if these lesions are clonally related or independently occurring primary malignancies. Herein we present a cohort (N = 22) with dual pancreatic ductal adenocarcinoma (PDAC) tumors to resolve previously conflicting reports and interrogate the underlying biological and clinical characteristics of this patient population. Next-generation sequencing of paired lesions (N = 10) revealed that while most dual PDAC are clonally related, independently occurring lesions do occur, irrespective of the interval between lesions. Integrated clinical, genomic, and histopathological analyses revealed a high frequency of lymph node-negative, intraductal papillary mucinous neoplasm (IPMN)-associated cancers, *KRAS* and/or *SMAD4* wild-type tumors, and classical subtype by immunohistochemistry, all collectively associated with more favorable outcomes. Acknowledging the highly selected nature of this cohort, isolated intrapancreatic metastases demonstrate a more indolent biology and these patients may benefit from personalized management approaches beyond traditional paradigms.

## Introduction

Pancreas cancer is an increasingly common malignancy and a leading cause of cancer-related morbidity and mortality^1,2^. Despite advances, long-term survival remains low with limited approved systemic treatment options^3^. Many groups, including ours, have published detailed pathological and genomic profiles of patients with pancreas adenocarcinoma (PDAC) and PDAC variants in order to refine our understanding of pancreas cancer and the distinct molecular and pathological subtypes with the goal of customizing treatment paradigms and improving outcomes for patients^4^. Individuals with PDAC who have two apparent independent primary lesions represent a unique subset of patients whose disease biology is not well understood. Dual pancreas lesions can occur synchronously, in which they appear both radiographically and pathologically distinct, or as metachronous lesions, with the second lesion occurring in a distant area of the remnant pancreas. Discerning the etiology of these lesions as related or independent lesions is critical to understanding the underlying biology and informing clinical decision-making, particularly in the emerging era of biomarker-directed therapeutics in pancreas cancer.

Limited prior case reports and small case series have resolved the etiology of these legions based on the time interval between lesions, histopathological features, and/or genomic profiling of oncogenic *KRAS* mutations^5–8^. Across these reported cases (*N* = 27 patients across *N* = 4 studies), 14 patients’ paired tumors were characterized as tumor recurrence, 9 as second primary malignancies, and 4 were deemed indeterminate with respect to relatedness. However, given that 90–95% of PDAC harbor oncogenic *KRAS* mutations, determining the etiology of these lesions based on histopathological features and *KRAS* allele alone is problematic^9^. *KRAS* driver mutations are acquired early in tumorigenesis and 90% of precursor low grade pancreatic intraepithelial neoplasia (PanIN)-1A and at least 60% of intraductal papillary mucinous neoplasms (IPMNs) have oncogenic *KRAS* mutations^10–12^. As a result, independent transformation events arising from the same precursor lesion may contain an identical oncogenic *KRAS* mutation. Furthermore, as almost 90% of oncogenic *KRAS* mutations in PDAC occur within only a limited conserved set of *KRAS* alleles at exon 2 codon 12 (G12D, G12V, or G12R), the probability of sharing mutant *KRAS* alleles is relatively high^13^. Lastly, intratumoral heterogeneity and tumoral evolution complicate these analyses, particularly for metachronous lesions which can be separated by several years and span anti-cancer therapies.

Only three studies with limited sample sizes have utilized current standard next-generation sequencing (NGS) to interrogate the etiology of these tumors^14–16^. While two of these studies concluded that all paired tumors (*N* = 3 synchronous, *N* = 4 metachronous) were clonally related^14,15^, one study identified 3 patients with independent primary tumors^16^. Here, in this detailed annotated series, we present the comprehensive clinical, pathological, and genomic features of the largest cohort reported to date (*N* = 22) to resolve these previous conflicting reports and interrogate the underlying biological and clinical characteristics of this rare subset of patients with dual PDAC.

## Results

### Patient characteristics

**Fig. 1:**
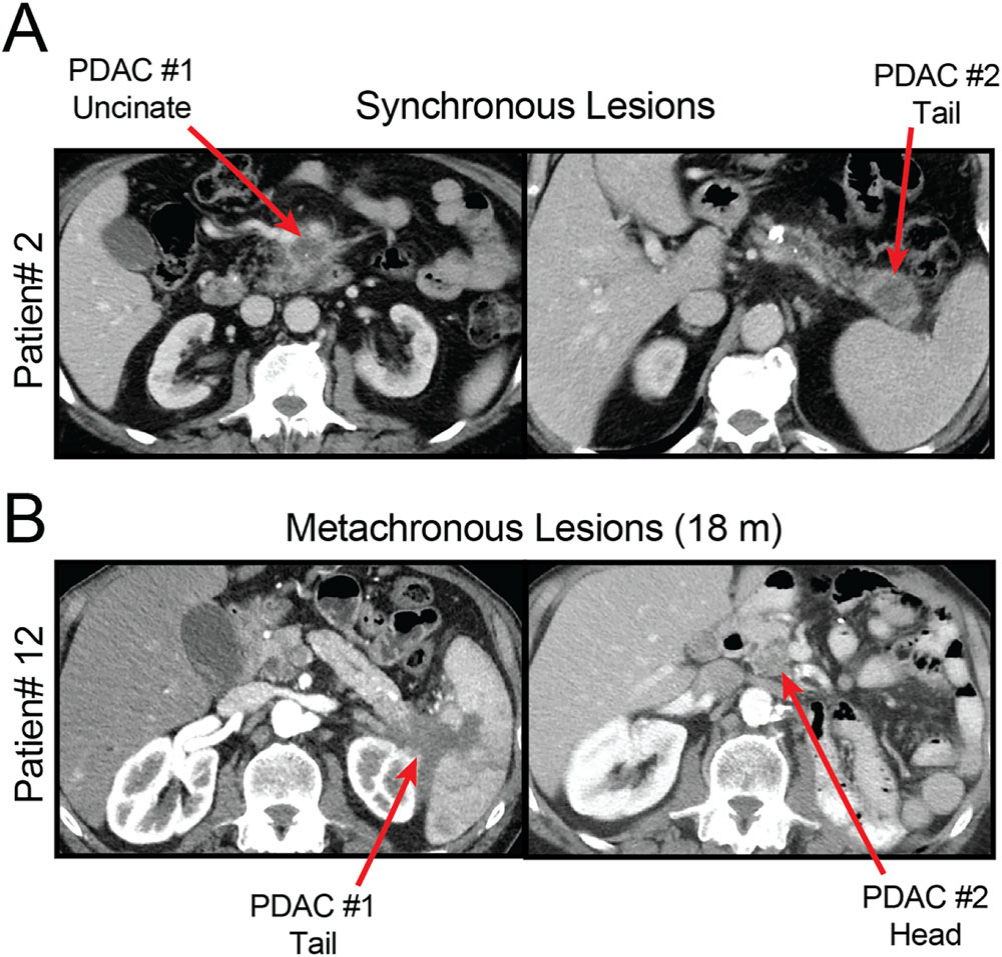
Synchronous and metachronous occurring PDAC. Representative computed tomography (CT) scans demonstrating **A** synchronously (patient 2) or **B** metachronously (patient 12; 18-month interval) occurring PDAC without radiographic connection.

Patients treated at Memorial Sloan Kettering Cancer Center (MSK) from 2008 to 2024 were retrospectively identified through review of institutional databases (Fig. S1). Participant lists were manually curated for patients with two invasive carcinomas of the exocrine pancreas. For synchronous lesions, participant inclusion required the absence of radiographic or pathologic (if resected) connection between the lesions (e.g., Fig. 1A). For metachronous lesions (e.g., Fig. 1B), patients were excluded if carcinoma was present at the pancreatic margin at the time of resection of their first pancreas cancer (PDAC1). A total of *N* = 22 patients were identified to have two independent appearing PDACs occurring either synchronously (*N* = 6) or metachronously (*N* = 16).

To understand whether patients with dual PDACs represent a patient population at elevated risk for development of PDAC, we first evaluated the cohort for established modifiable and non-modifiable PDAC risk factors (Table 1). The median age at diagnosis of PDAC1 was 68 years (range: 51–79), 50% were female, and 91% were Caucasian. We considered multiple risk factors at the time of diagnosis of PDAC1 including weight (body mass index (BMI) > 30), personal history of tobacco use, pre-existing diabetes mellitus, history of pancreatitis, or known pre-malignant pancreatic lesion. We examined both personal history of a non-pancreatic malignancy and family history of a first degree relative with a diagnosis of PDAC. No patient in this cohort had an exposure history to known occupational carcinogens linked with PDAC pathogenesis. Across the cohort, the prevalence of PDAC risk factors within this cohort are similar to those reported in the general PDAC population^17–21^. Furthermore, there were no significant differences in PDAC risk factors between patients with synchronous or metachronous lesions (Table 1).

**Table 1:** Baseline Patient Demographics and PDAC Risk Factors

Characteristic	Overall, N = 22^a^	Synchronous, N = 6^a^	Metachronous, N = 16^a^	p-value^b^
Age (PDAC1)	68 (63, 74)	74 (71, 75)	68 (61, 71)	0.070
Age (PDAC2)	72 (66, 75)	74 (71, 75)	70 (65, 75)	0.4
Interval PDAC1to PDAC2 (m)	--	0 (0, 1)	33 (21, 73)	<0.001
Female Sex	11 (50%)	2 (33%)	9 (56%)	0.6
Race				0.5
Caucasian	20 (91%)	5 (83%)	15 (94%)	
Asian	2 (9.1%)	1 (17%)	1 (6.3%)	
BMI at Diagnosis of PDAC1	23.9 (22.4, 25.8)	24.6 (23.5, 26.6)	23.8 (22.3, 25.1)	0.7
Unknown	2	1	1	
First Degree Relative with PDAC	3 (14%)	1 (17%)	2 (13%)	>0.9
Personal History of non-PDAC Malignancy	7 (32%)	2 (33%)	5 (31%)	>0.9
History of Recurrent Pancreatitis	1 (4.8%)	0 (0%)	1 (6.7%)	>0.9
Unknown	1	0	1	
Known Pre-Malignant Pancreatic Lesion	2 (9.5%)	0 (0%)	2 (13%)	>0.9
Unknown	1	0	1	
History of Tobacco Use	13 (59%)	3 (50%)	10 (63%)	0.7
History of Pre-Surgical Diabetes	5 (23%)	3 (50%)	2 (13%)	0.10
Germline Pathogenic Variant				
*gATM*	2 (13%)	2 (50%)	0 (0%)	0.05
Unknown	6	2	4	

^a^Median (IQR); n (%).

^b^Wilcoxon rank sum exact test, Fisher’s exact test, Wilcoxon rank sum test.

m: months, *gATM* germline pathogenic variant in Ataxia-telangiectasia mutated.

Approximately 10–15% of PDAC occurs in the context of a pathogenic germline variant associated with a known cancer susceptibility syndrome^22^. Similarly, among patients with available germline profiling (*N* = 16), we identified 2 (13%) patients carrying pathogenic germline variants, both in the *ataxia telangiectasia mutated (ATM)* gene (Table 1). Both patients with identified germline variants presented with synchronous lesions (50%, 2/4). Interestingly, none of the 12 patients with available germline profiling who presented with metachronous lesions were found to harbor pathogenic germline variants. Expansion of germline profiling beyond known PDAC susceptibility genes did not identify any additional pathogenic variants.

### Clinical and pathological characteristics

**Fig. 2:**
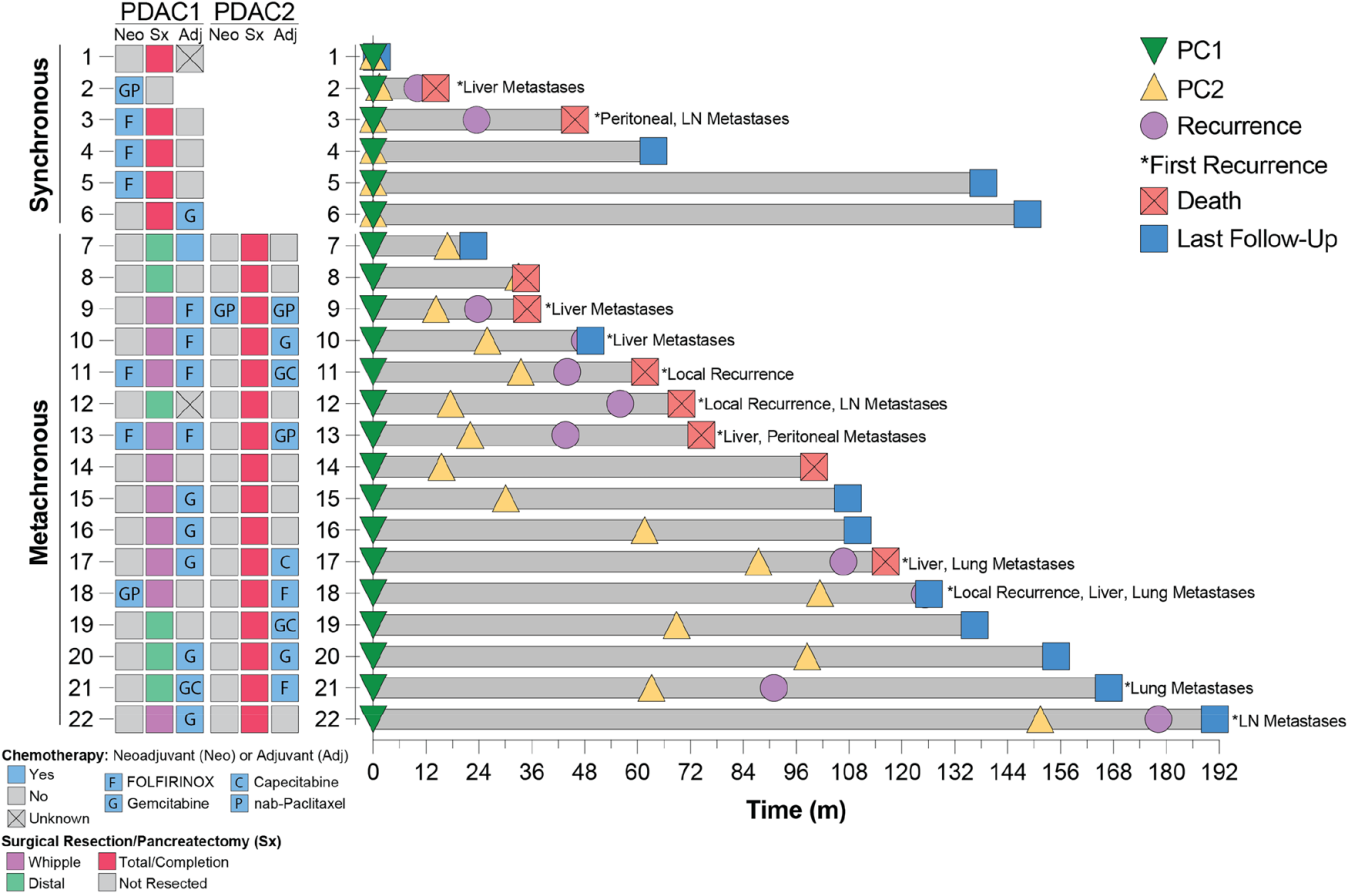
Clinical course of patients with synchronous or metachronous PDAC. Summary of treatment history and clinical course for patients with synchronous (patients 1–6) and metachronous (patients 7-22) dual PDAC. Only treatment in regard to PDAC1 or PDAC2, and not for any subsequent recurrence, is presented. Administration of chemotherapy, regardless of the duration, is denoted as ‘yes’. Swimmer’s plot detailing the patients’ clinical course highlights the diagnosis of PDAC1 (green triangle) and PDAC2 (yellow triangle), disease recurrence (purple circle), site of first subsequent disease recurrence (*; LN = lymph node), and either death (red square) or last follow-up (blue square).

The majority of patients (4/6, 67%) with synchronous lesions received neoadjuvant chemotherapy and 83% (5/6) underwent resection with total pancreatectomy (Fig. 2, Table 2, Table S1). All patients with synchronous lesions who underwent resection had negative margins (R0 resection) for both tumors. One patient did not undergo resection due to persistent locally advanced, unresectable disease following neoadjuvant therapy (patient 2). Pathologic staging of individual resected lesions identified that 40% (2/5) of lesions in the proximal pancreas (PDAC1) and 60% (4/5) lesions in the distal pancreas (PDAC2) were T1 or demonstrated a pathologic complete response (pCR) to neoadjuvant treatment and 40% (2/5) of patients had lymph node-negative (N0) disease (Table 2). All evaluable patients (5/5) received either neoadjuvant and/or adjuvant systemic therapy. At a median follow-up of 99.7 m (range 0.9–146.4 m), two patients with synchronous lesions had recurrent/progressive disease (1/5 of resected patients), both of whom have died (Fig. 2). This resulted in 1-, 2-, and 3-year event free survival (EFS) rates of 80% [95% CI: 52–100%], 60% [95% CI: 29–100%], and 60% [95% CI: 29–100%] and 1-, 2-, and 3-year overall survival (OS) rates of 100%, 80% [95% CI: 52–100%], and 60% [95% CI: 29–100%], respectively (Fig. S2A).

**Table 2:** Clinical Characteristics

Synchronous (N = 6)	Proximal^a^	Distal^a^
Neoadjuvant Chemotherapy	4 (67%)	
Resection	5 (83%)	
Pathological T Stage (N = 5)		
pCR	0 (0%)	1 (20%)
T1/ypT1	2 (40%)	2 (40%)
T2/ypT2	2 (40%)	2 (40%)
T3/ypT3	1 (20%)	0 (0%)
Pathological N Stage (N = 5)		
pN0/ypN0	2 (40%)	
pN1/ypN1	3 (60%)	
Adjuvant Chemotherapy	1 (25%)	
Unknown	2	
Neoadjuvant or Adjuvant Chemotherapy	5 (100%)	
Unknown	1	
**Metachronous (N** = **16)**	**PDAC1**^a^	**PDAC2**^a^
Location		
Head/Uncinate	10 (63%)	6 (38%)
Body/Tail	6 (38%)	10 (63%)
Neoadjuvant Chemotherapy	3 (19%)	1 (6%)
Resection	16 (100%)	16 (100%)
Pathological TNM Stage		
pCR^b^	1 (6%)	0 (0%)
IA/B	12 (75%)	9 (56%)
IIA/B	3 (19%)	6 (38%)
III	0 (0%)	1 (6%)
Adjuvant Chemotherapy	11 (73%)	9 (56%)
Unknown	1	--
Neoadjuvant or Adjuvant Chemotherapy	12 (80%)	9 (56%)
Unknown	1	--

^a^n (%).

^b^pCR = 1/3 (33%) for patients with metachronous tumors who received neoadjuvant chemotherapy.

Among the 16 patients with metachronous lesions, PDAC1 more commonly occurred in the proximal pancreas (63%, 10/16) than the distal pancreas (38%, 6/16) (Fig. 2, Table 2, Table S1). Only 19% (3/16) of patients with metachronous lesions received neoadjuvant chemotherapy for PDAC1. Most (15/16, 94%) underwent optimal surgical resection (R0) for PDAC1 with one patient who underwent a distal pancreatectomy with a positive posterior soft tissue margin and negative pancreatic parenchymal margin (patient 20). Pathologically, 81% (13/16) of PDAC1 were stage I or demonstrated a pCR to neoadjuvant therapy. Only two patients (12%, 2/16) had node positive (pN1) disease on final pathology for PDAC1. Most (80%, 12/15) patients received either neoadjuvant and/or adjuvant systemic treatment for PDAC1. Median interval from PDAC1 to PDAC2 was 33 months (range: 14–149 m). Just 1 of 16 patients (6%) received neoadjuvant chemotherapy for PDAC2 and all patients (16/16) underwent completion pancreatectomy. Only 57% (9/16) of patients with metachronous PDAC received either neoadjuvant and/or adjuvant systemic therapy for PDAC2. At a median follow-up of 47.6 m (range: 5.8–102.3 m) from PDAC2, 10 patients have recurrent disease, and 7 patients have died resulting in 1-, 2-, and 3-year EFS rates of 80% [95% CI: 63–100%], 54% [95% CI: 33–86%], and 40% [95% CI: 22–75%] and 1-, 2-, and 3-year OS rates of 94% [95% CI: 83–100%], 87% [95% CI: 72–100%], and 71% [95% CI: 51–100%],respectively (Fig. S2B). These lengthy survival outcomes are particularly notable given the extended interval from PDAC1 to PDAC2.

Most patients across both cohorts (95%, 21/22), underwent a total or completion pancreatectomy. At a median follow-up of 55.7 m (range 0.9—146.4 m) from PDAC2, 11 patients had recurrent/metastatic disease resulting in 1-, 2-, and 3-year EFS rates of 80% [95% CI: 65–100%], 55% [95% CI: 37–82%], and 45% [95% CI: 28–73%] and 1-, 2-, and 3-year OS rates of 95% [95% CI: 87–100%], 85% [95% CI: 71–100%], and 68% [95% CI: 50–93%], respectively (Fig. S2C).

### Histopathologic review reveals similarity across paired PDAC

Histopathologic review of paired tumors revealed two distinct invasive carcinomas with similar morphologic features for both the invasive carcinoma and associated precursor lesions in the majority of patients (Table S1). All six patients with synchronous tumors had conventional type PDACs. For one patient (patient 1), both PDAC1 and PDAC2 were associated with a precursor lesion, a pancreaticobiliary type intraductal papillary mucinous neoplasm (IPMN). For two additional patients, both PDACs were associated with pancreatic intraepithelial neoplasia (PanIN). In the remaining three cases, assessment of paired precursor lesions was not possible because at least one specimen was a biopsy. For 14 of 16 patients with metachronous tumors (88%), both carcinomas were either conventional-type PDAC or showed tubular (PDAC-like) invasion. Precursor lesions were distributed as follows: six patients had PanIN associated with both carcinomas; two had IPMN associated with both carcinomas; two had intraductal tubulopapillary neoplasms (ITPNs) associated with both carcinomas; three had an IPMN associated with one carcinoma and PanIN with the other; and in three additional cases, one carcinoma was associated with PanIN while the other could not be evaluated due to incomplete slide availability. Only two patients (2/22, 9%; Patients 7 and 8) had morphologically distinct paired tumors (colloid carcinoma vs conventional-type PDAC).Only two patients’ (2/22, 9%, patients 7 and 8) had morphologically distinct paired tumors (colloid carcinoma *vs*. conventional type PDAC). Immunohistochemical (IHC) analysis of available paired lesions (*N* = 22 tumors from *N* = 14 patients) revealed that the PDACs in all but one patient had a classical phenotype, characterized by GATA6 expression and absent p40 and cytokeratin 5/6 (CK5/6) staining (Table S1; see Methods). For one patient (patient 9), IHC revealed a mixed phenotype characterized by GATA6 and CK5/6 expression with absent p40 staining.

### Somatic profiling reveals most, but not all, paired PDAC are clonally related

All tumors for which there was sufficient genomic DNA (gDNA) underwent somatic profiling *via* MSK-IMPACT, a hybridization capture based NGS assay for targeted deep sequencing of all exons and selected introns of up to 505 cancer associated genes (29/44 tumors across 19 patients)^23^. *RAS* wildtype (WT) tumors with sufficient available RNA (N = 1) were reflexed for oncogenic fusions by targeted RNA sequencing.

**Fig. 3:**
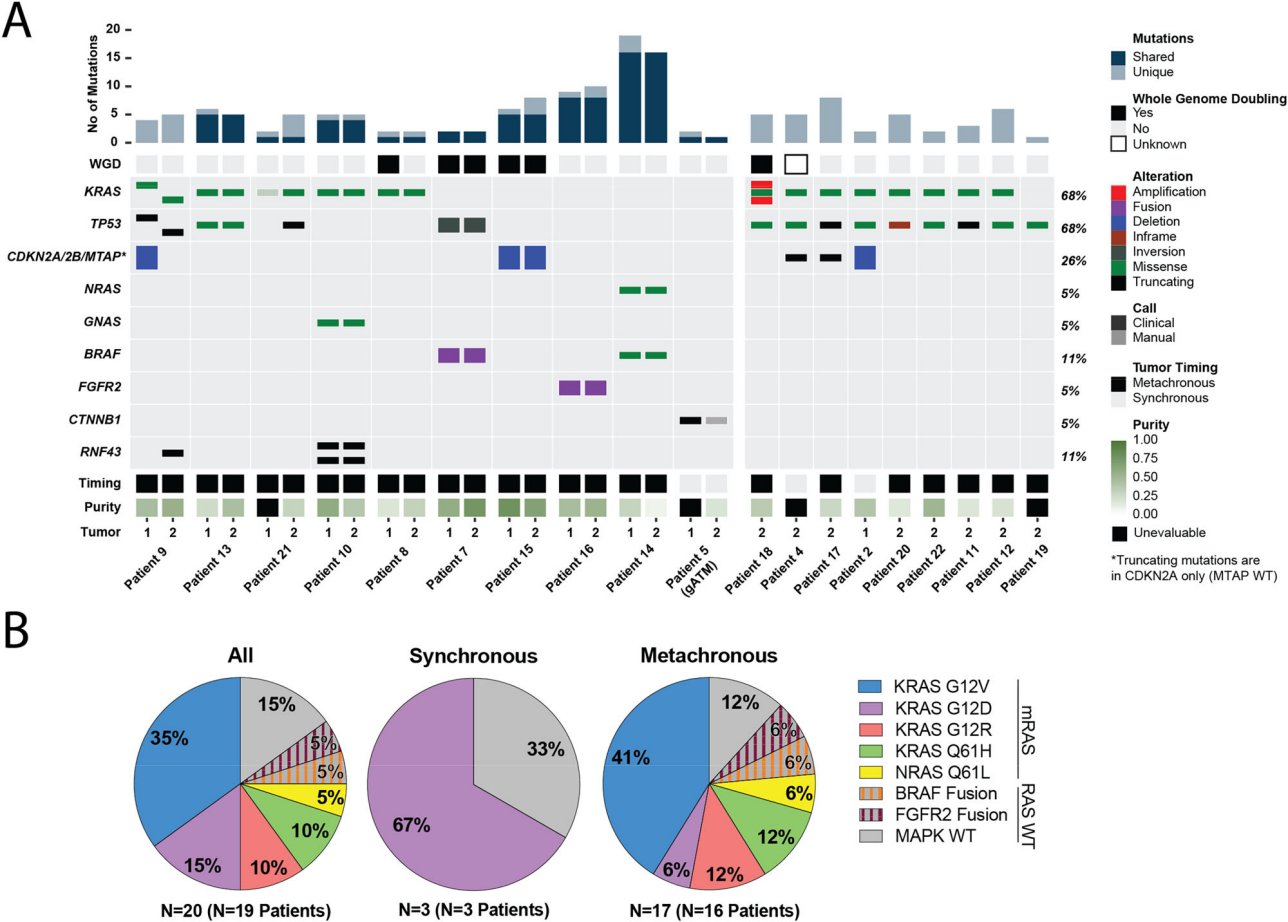
Somatic alterations of synchronous and metachronous occurring PDAC. **A** Oncoprint of somatic driver alterations identified by MSK-IMPACT. *RAS* WT tumors profiled by MSK-FUSION for oncogenic fusions. Samples grouped by paired or single tumor profiling availability with cohort-level alteration frequencies calculated at the patient level. For paired tumors only, notched mutations in the same gene represent different alleles; mutations identified in one tumor meeting clinical reporting thresholds were manually genotyped to evaluate for low frequency alterations in the paired tumor. For *CDKN2A/B/MTAP*, truncating mutations are in *CDKN2A* only (*MTAP* WT). **B** Distribution of *RAS* and *MAPK* alterations identified. For patient 9, clonally independent tumors are individually represented.

NGS was performed on both PDAC1 and PDAC2 for 10 patients (45%), including one (17%) with synchronous tumors and nine (56%) with metachronous tumors, allowing us to investigate relatedness of the paired lesions (Fig. 3A). In addition to alterations which met clinical reporting thresholds, paired samples were genotyped for any mutations identified in only one tumor to account for low frequency mutations in the paired tumor. Across the 20 paired tumors, a median of 5 mutations (range: 1-19) were detected with only 3% identified through genotyping. For six of ten patients (patients 7,10,13,14,15,16), paired metachronously occurring lesions are recognizably clonally related with at least 56% mutations shared (range: 56–100%), including 100% of PDAC driver mutations. Furthermore, paired tumors from all six of those patients had sufficient purity ( > 10%) to be assessed for copy number alterations (CNA) (Fig. S4). Three patients (patients 7, 13, 15) shared 100% of the oncogenic gene-level CNAs. While no oncogenic gene-level events were identified for patient 14, paired tumors had similar CNA profiles and shared 84% of the identified mutations confirming their relatedness. Though PDAC2 of patient 16 harbored exclusive oncogenic gene-level homozygous deletions not found in PDAC1, the tumors had otherwise largely overlapping CNA profiles and the tumors shared all PDAC driver mutations confirming their relatedness. No copy number events were observed in either tumor from patient 10.

For three patients, paired tumors only shared one somatic alteration (patients 5, 8, and 21). Given the low purity of both tumors, relatedness of PDAC1 and PDAC2 was difficult to assess for patient 8. However, due to a shared *KRAS* G12V mutation and an overlapping CNA profile (chromosomes 2, 3, 9, 17, and 21), we conclude that these lesions were likely clonally related (Fig. S4). Similarly, for patient 5, only one of two mutations identified by NGS were shared across PDAC1 and PDAC2, a *CTNNB1* loss-of-function mutation which is uncommon and not a known driver of conventional PDAC. Given the synchronous nature of these tumors without interval time or treatment, a similar CNA profile, and a shared rare mutation in PDAC, we again propose that these tumors are related.

NGS did not identify shared mutations or CNAs meeting clinical reporting thresholds for patient 21, likely due to low purity of PDAC1. Manual genotyping identified one read (1/455, VAF: 0.22%) in PDAC1 supporting a shared *KRAS* G12D mutation identified in PDAC2 without any evidence of other mutations found in PDAC2. As the identification of the *KRAS* G12D mutation in PDAC1 is below the error rate of NGS, and manual genotyping of the *KRAS* G12 locus of PDAC1 also identified 7 reads supporting G12R and 1 read supporting G12C mutations, we sought to clarify the relatedness of these lesions by ddPCR (Fig. S5, Table S2). While ddPCR supported the enrichment of *KRAS* G12R (VAF 1.6%) over G12D (VAF 0.44%) mutations, PDAC1 was also found to harbor both the *TP53* E326* (VAF 0.72%) and *FBXW7* R505C (VAF 0.47%) mutations identified in PDAC2 demonstrating that these tumors are clonally related. The etiology of the *KRAS* G12R mutation, also identified in PDAC2 at low VAF by ddPCR (0.09%, 3 droplets), remains unclear (see Discussion).

For patient 9, NGS of their paired tumors revealed no shared mutations, including disparate *KRAS* (PDAC1: Q61H, PDAC2: G12V) and *TP53* mutations, exclusive deletion of *CDKN2A* in PDAC1, and largely independent CNA profiles. We therefore concluded that this patient’s metachronous PDACs, occurring 14 months apart, are clonally independent. We used ddPCR to confirm the absence of a *KRAS* Q61H mutation in PDAC2 but there was a single positive droplet (VAF 0.04%) for *KRAS* G12V in PDAC1 (Table S2). However, it is worth nothing that both PDAC1 and PDAC2 had low levels of G12R (PDAC1: 0.04%, PDAC2: 0.37%) and PDAC2 had evidence of G12D (0.08%), again bringing into question the etiology and clinical significance of low VAF *KRAS* mutations. Indeed, for all patients tested by ddPCR, multiple non-NGS-detected KRAS alleles were identified, all with VAF < 2% (Fig. S5, Table S2).

Two additional patients (patients 2, 18) did not have sufficient tissue quality to perform NGS on both samples but were sufficient for an orthogonal approach to test *KRAS* status (*N* = 1 *via* Idylla^TM^, *N* = 1 *via* ddPCR). For these two patients, NGS of one tumor and *KRAS* profiling of the other tumor reveled identical *KRAS* alleles suggesting, but not confirming, that paired tumors for these patients were also clonally related. Importantly, the limit of detection of Idylla^TM^ for *KRAS* G12R is 5% VAF and the VAF from ddPCR of PDAC2 from patient 2 was 24%, both far above the frequency of low VAF *KRAS* alleles of unclear etiology^24^.

### Pan-cohort oncogenic drivers and co-mutational profiles

Following the adjudication of PDAC relatedness, NGS of all available tumors (*N* = 29 tumors) from 19 patients (86%) were analyzed to interrogate the underlying genomics of this rare subset of patients (Fig. 3A). No somatic profiling was available for three patients (14%; patients 1, 3, 6). Frequencies of oncogenic drivers and co-alterations were quantified at the patient level. Given the unclear significance of low VAF *KRAS* mutations detected by ddPCR, only *KRAS* alleles detected by NGS and meeting clinical reporting thresholds were included.

Oncogenic *KRAS* mutations (m*KRAS*) were identified in 13 (68%) patients with one additional patient each identified to harbor an oncogenic *NRAS* mutation, *BRAF* fusion, or *FGFR2* fusion (Fig. 3B). No oncogenic mutations in the mitogen-activated protein kinase (MAPK) pathway were identified in three patients (16%). We considered all mutations in *mRAS* tumors, accounting for patient 9’s unrelated tumors independently (*N* = 15 tumors from 14 patients), and found seven (47%) with *KRAS* G12V, three (20%) with *KRAS* G12D, two (13%) with *KRAS* G12R, two (13%) with *KRAS* Q61H, and one (7%) with *NRAS* Q61L mutations. Among the three patients with synchronous lesions, two (67%) were *KRAS* G12D mutated while no oncogenic mutation was identified for one (33%). Across patients with metachronous occurring PDAC (*N* = 16), *KRAS* WT tumors accounted for 29% (*N* = 5/17) while among metachronous occurring m*RAS* tumors (76%; *N* = 13 tumors from 12 patients), seven (54%) *KRAS* G12V, two (15%) *KRAS* G12R, two (15%) *KRAS* Q61H, one (8%) *KRAS* G12D, and one (8%) *NRAS* Q61L mutations were identified.

We next examined the patient-level frequencies of oncogenic alterations in our cohort. Interestingly, *KRAS* and *MAPK* WT tumors accounted for 32% and 13% of cases, respectively. Among metachronous m*RAS*, *KRAS* G12V mutations were the most frequently observed (58%), while *KRAS* G12D mutations were less common (8%). *TP53* mutations were identified in 68% of cases, and *CDKN2A/B* alterations in 26%. Notably, no *SMAD4* alterations were detected across the entire cohort.

To contextualize these findings, we reviewed data from a recently reported cohort of patients with resected PDAC profiled by MSK-IMPACT (*N* = 620), providing a reference for genomic features commonly observed in the broader resected PDAC population^4^. Among the 620 patients, only 5% and 2% of tumors were *KRAS* or *MAPK* WT, respectively. Interestingly, even after matching their methodology (see Methods^4^) and thereby enhancing the detection of sub-threshold *KRAS* mutations, we still identified 26% *KRAS* WT tumors across the cohort presented here. Among m*RAS* tumors (*N* = 586), 34% were *KRAS* G12V mutated while 39% were *KRAS* G12D mutated^4^. Lastly, 23% of tumors harbored SMAD4 alterations in contrast to 0% identified in the cohort presented here^4^.

## Discussion

Herein we present the comprehensive clinical, pathologic, and genomic features of patients with two independent-appearing PDACs to determine the relatedness of the lesions, interrogate the underlying biologic features of this distinct patient subset, and inform prognostication. Our data clearly demonstrate that clinical and pathologic features alone are insufficient to reliably resolve the relatedness of paired lesions. Additionally, while the dominant *KRAS* allele was the most important deciding factor, NGS is required for optimal determination given a high frequency of *KRAS* WT tumors in this cohort of patients. With an updated understanding of the factors required to define the relatedness of lesions, summary and re-adjudication of cases published in the literature are presented in Table S3. Overall, the cohort presented herein supports the observation that dual PDACs are most often clonally related. However, clonally independent dual PDACs do occasionally occur (patient 9).

Patients were excluded from this study if synchronous lesions were identified to have direct radiographic or pathologic communication or if metachronous lesions occurred in the setting of a positive pancreatic margin following resection of PDAC1. Despite careful exclusion of patients at elevated risk for local recurrence, most cases reported herein are clonally related, representing intrapancreatic spread. Intraparenchymal spread of PDAC has been well described and may occur through intrapancreatic metastasis or direct extension, thought to largely occur *via* the pancreatic ducts^25–28^. Consistent with the latter, eight patients (36%) presented here had disease that arose in the context of an intraductal precursor neoplasm. All six of these patients interrogated by NGS had clonally related lesions.

Paired NGS of synchronously occurring lesions presented here (*N* = 1) and by Connor et al. (2019; *N* = 3) revealed that all lesions are clonally related^15^. Additionally, for patient 2, NGS of PDAC1 had an identical *KRAS* mutation in PDAC2 by ddPCR (VAF 24%) suggesting, but not confirming, that these lesions are clonally related. In contrast, Fujita and colleagues (2020) identified clonally independent lesions for three of four patients presenting with synchronous PDAC^16^ consistent with recent reports in synchronous colon, non-small cell lung (NSCLC), and head and neck squamous cell (HNSCC) cancers in which synchronous occurring lesions assayed by NGS were found to be largely clonally independent and in support of the concept of ‘field cancerization’^29–32^. It is particularly interesting that 60% (3/5) of patients with synchronous occurring lesions presented here and in the literature for whom germline profiling was available harbored a germline variant in *ATM* (*N* = 2) or *PALB2* (*N* = 1), which are associated with accelerated rates of malignant transformation, were found to have clonally related tumors. In synchronous ovarian endometroid and endometrial carcinomas, independent primaries were exclusively found in patients with Lynch Syndrome and not in sporadic occurring cancers^33,34^. Of note, both our study and Connor et al.^15^ included a North American patient population while Fuijita et al.^16^ examined a Japanese patient population; whether the difference in conclusions is a result of small sample sizes across all studies or confounding environmental or genetic factors remains unclear.

Similar to patients with synchronous occurring lesions, metachronous occurring lesions presented here (N = 9) and in the literature (*N* = 6; Table S3) for whom NGS of paired lesions is available are mostly (N = 13/15, 87%) clonally related. However, clonally independent lesions do occur (e.g., patient 9). Importantly, lesion relatedness appears independent of the interval between PDAC1 and PDAC2; the clonally independent lesions occurred over one of the shortest intervals (14 months) while clonally related lesions occurred with intervals of over 10 years from PDAC1. Similarly, our group recently reported on another patient with PDAC who developed distant metastatic disease 18 months following resection who was found to have an intrapancreatic ‘recurrence’ at autopsy^35^. NGS of the intrapancreatic and metastatic ‘recurrences’ confirmed that these lesions were clonally independent from the original resected primary tumor. We therefore conclude that independent primaries can indeed occur within relatively short intervals. These findings strongly support confirmation of the presence of any actionable alteration prior to administration of targeted therapies in any context where there is the possibility for a second primary. This is of particular importance in the emerging era of targeted therapies in PDAC, most notably KRAS directed therapies.

Interestingly, no individual with metachronous occurring lesions presented here (*N* = 12) or in the literature (N = 1) for whom profiling was available harbored a germline alteration in any known PDAC predisposition gene, including the individual patient with clonally independent tumors. Patient 9 was a current every-day smoker up until the time of diagnosis of PDAC2 (40-50 pack year history), had a prior personal history of breast cancer, and this patient’s mother had a history of both pancreas and breast cancers, suggesting that additional environmental and genetic factors may have contributed to accelerated rate of transformation.

Beyond determining the relatedness of the lesions, in contrast to prior reports, the size of our cohort offers novel insights into the underlying biology of this relatively rare subset of PDAC. Consistent with our finding that most lesions are clonally related, our patient population presented with similar rates of PDAC risk factors to the general PDAC population, suggesting that this patient population is not at an increased risk for the development of PDAC. While the relatively small size of our cohort prevented statistical testing, in comparison to the general population of patients with PDAC, integrated genomic and histopathological analyses revealed a high frequency of lymph node negative disease, IPMN-associated cancers, *KRAS* and *SMAD4* wild-type tumors, and classical PDAC subtype by IHC, all of which are associated with more indolent biology and favorable outcomes^4,36–41^. In contrast, for patient 9, whose tumors were clonally independent, both tumors were node-positive, not associated with an intraductal lesion, *KRAS* and *TP53* mutated, and were the only cases tested to demonstrate a mixed basal/classical phenotype by IHC more consistent with the general population of patients with PDAC.

Among m*RAS* metachronous occurring tumors we identified 58% G12V mutations and only 8% G12D mutations as compared to the general population of patients with resectable PDAC who were found to harbor 34% G12V and 39% G12D mutations^4^. Across several large data sets from our group and others, *KRAS* G12V alterations have been associated with fewer inactivating mutations in tumor suppressor genes and improved outcomes as compared to *KRAS* G12D mutated tumors^4,42,43^. Furthermore, while we detected the predicted frequencies of *TP53* and *CDKN2A/B* alterations, no *SMAD4* alterations were identified in this cohort in contrast to the more than 20% frequency of *SMAD4* mutations seen across PDAC stages^4,42^. *SMAD4* loss has been associated with PDAC progression and metastasis again supporting the enrichment of favorable genetics in this cohort which may promote more indolent, locally recurrent disease over metastatic potential^36,44^.

Interestingly, for all tumors tested, multiple *KRAS* hotspot mutations were identified by ddPCR, including patients who had a dominant *KRAS* allele identified by NGS. While clinically called mutations had a ddPCR VAF > 10%, all other detected mutations had a median VAF of 0.14% (range: 0.01–1.8%). The etiology of these mutations remains unclear as either small independent malignant clones, sub-clonal second *KRAS* mutations within the dominant clone, or bystander mutations from neighboring pre-malignant lesions incorporated into the malignant clone by bulk gDNA extraction. We favor the latter explanation for several reasons: 1) normal human pancreas glands contain hundreds of PanINs, the majority of which harbor ≥ 1 *KRAS* mutation^10,45^, 2) while single-cell DNA sequencing of human PDAC has identified rare tumors or even cells with > 1 *KRAS* mutation, the authors found no correlation with other driver mutations suggesting that these cells were not part of the malignant clone^46^, and 3) emerging data from patients receiving allele specific KRAS inhibitors have revealed second *KRAS* mutations as only a rare mechanism of acquired treatment resistance^47^. Collectively based on these data, we consider PDAC1 and PDAC2 of patient 9 to be independent despite sharing low level *KRAS* mutations identified by ddPCR. Further analysis of pre- and post-KRAS targeted therapy specimens are needed to determine if emergent secondary *KRAS* alleles were previously present or represent de novo mutations. Furthermore, whether low VAF *KRAS* mutations ( < 2%) detected by orthogonal tumor-based profiling are clinically actionable in NGS-designated *KRAS* WT tumors remains unclear.

Despite the detection of multiple low VAF *KRAS* mutations by ddPCR, we conclude that tumors from 32% of patients are, for all clinical intent and purpose, *KRAS* WT tumors as compared to the 4–12% reported of patients with *KRAS* WT resected PDAC^42^. As is guideline endorsed, we recommend NGS for identification of oncogenic *KRAS* mutations and other actionable alterations independent of the disease stage in patients with conventional PDAC or PDAC variants^48^. Importantly, identification of actionable mutations and administration of matched targeted therapies has been associated with improved survival in PDAC^49^. For patients with limited tumor material for which NGS is not feasible, we recommend testing for *KRAS* mutations using an orthogonal tumor-based approach to evaluate for hotspot mutations and/or evaluation of circulating tumor DNA (ctDNA) which may also allow for detection of additional actionable alterations. Identification of oncogenic *RAS* mutations is increasingly important given the emerging KRAS and pan-RAS inhibitors rapidly progressing through clinical development (e.g., RASolute 302, NCT06625320). As oncogenic *KRAS* mutations are thought to be acquired early in PDAC tumorigenesis and clonal in nature, we recommend caution in overinterpretation of the clinical actionability of low VAF *KRAS* alleles ( < 2%) identified *via* somatic profiling, particularly those not detected by clinically validated NGS methods. This theoretical threshold is significantly lower for *KRAS* alterations detected in ctDNA, likely in part due to increased burden and shedding of malignant clones^50^. For tumors in which oncogenic mutations in the MAPK pathway are not identified, as highlighted here by the identification of an oncogenic and clinically actionable *FGFR2* fusion (patient 16), we recommend targeted RNA sequencing for the presence of actionable fusions which are known to be enriched in this population^4,37,38,51,52^.

While the retrospective design of this study with a relatively small, highly selected, sample size prevents us from statistically contextualizing the clinical outcomes for this cohort of patients, underpinned by the favorable disease biology discussed above, patients with isolated pancreatic metastases demonstrate relatively favorable outcomes compared to the general population of patients with resected PDAC and benefit from personalized, multidisciplinary management approaches beyond traditional paradigms. For patients presenting with resectable, synchronous tumors, multidisciplinary discussion is vital to guide the sequencing of chemotherapy and surgery as well as the decision to offer a partial *vs*. total pancreatectomy. In general, as these tumors either represent isolated intrapancreatic metastases or multicentric carcinogenesis and the patient would be at high risk for intrapancreatic recurrence or another independent primary, respectively, total pancreatectomy is a reasonable upfront surgical approach for this rare subset of patients when deemed safe and feasible in light of the patient’s overall health. As no biomarkers for metachronous, independent PDAC or isolated intrapancreatic metastasis have been identified, these patients should be managed as the general PDAC population at the time of initial presentation. As the majority of metachronous tumors represent recurrent disease, our general multidisciplinary decision-making approach integrates the patient’s overall health, disease-free interval from PDAC1, and radiographic and biochemical data to guide the sequencing of systemic therapy and surgery.

Limitations of this study include the single institution, retrospective design, a primarily Caucasian demographic, lack of paired NGS for 55% (12/22) of patients, and low tumor purity with bulk gDNA extraction. Notable strengths include the size of the cohort of this relatively rare population of PDAC patients and the in-depth, comprehensive clinical, genomic, and pathological review.

## Methods

### Patient Selection, Clinical Data Collection, and Statistical Analyses

Patients from Memorial Sloan Kettering Cancer Center (MSK) were accrued retrospectively as approved by our Institutional Review Board. Patients were identified through review of institutional databases (Fig. S1). Specifically, the Hepatopancreaticobiliary Surgery Division patient database was reviewed for all patients who had undergone completion or total pancreatectomy or whose pancreatectomy specimen contained more than one pathology since 2012. Participant lists were then manually curated for patients with two invasive carcinomas of the exocrine pancreas; patients with at least one non-invasive lesion or non-exocrine pancreatic carcinoma histology were excluded. Patients with known, radiographically evident, precursor lesions were allowed. For synchronous lesions, participant inclusion required the absence of radiographic or pathologic (if resected) connection between the lesions. For metachronous lesions, patients were excluded if there was invasive carcinoma or non-invasive carcinoma present at the pancreatic margin at the time of resection of PDAC1; precursor lesions with any degree of dysplasia at the resection margin were allowed. Detailed demographic and clinical history were abstracted from the medical records. Clinical data were last updated on June 19, 2024.

Baseline characteristics ascertained at the time of diagnosis of PDAC1 were compared between synchronous *vs*. metachronous cases using Fisher’s exact test for categorical variables and Wilcoxon Rank-Sum test for continuous variables. Risk factors of personal history of a non-PDAC malignancy or family history of first degree relative with PDAC were collected at any timepoint from the medical record. Overall survival (OS) was calculated from date of PDAC2 until date of last follow up or death. EFS is defined as the time from diagnosis of PDAC2 until subsequent disease recurrence, progression, or death, whichever occurred first. OS and EFS were estimated using Kaplan-Meier methods and compared between patients with synchronous vs metachronous dual PDAC using log-rank test. All statistical analyses were performed using R Version 4.3.2. All *P-value* were two sided and *p-values* of < 0.05 were considered to indicate statistical significance.

### Histopathological Review and Immunohistochemistry

Available gross photographs and descriptions as well as all histologic sections were re-evaluated to confirm the diagnosis and further characterize the spectrum of morphology by using the current WHO criteria for pancreatic neoplasms^53^. Representative formalin-fixed paraffin-embedded (FFPE) tissue sections of each case for which paired paraffin blocks or unstained sections were available, were immunolabeled using the standard avidin-biotin peroxidase method. GATA6 (clone: D61E4, 1:500 dilution, Cell Signaling; RRID:AB_10705521), p40 (clone: BC28, 1:250 dilution, BioCare; RRID:AB_2858274) and CK5/6 (clone: D5/16B4, 1:400 dilution, Dako; RRID:AB_2281083) immunohistochemical stains were performed.

Two gastrointestinal/pancreatobiliary pathologists, blinded to clinical data, independently scored all stains on whole-slide images. For each marker, the percentage of positive tumor cells was recorded, and staining intensity was graded as weak (+), moderate (++), or strong (+++). Discordances were resolved by joint review. For GATA6, positivity was defined as ≥50% of tumor nuclei with moderate (++) or strong (+++) staining or ≥10% of tumor nuclei with strong (+++) staining. For p40, positivity was defined as ≥10% of tumor nuclei with moderate (++) or strong (+++) staining. For both GATA6 and p40, negative staining was defined as weak-only staining or <10% of nuclei positive. For CK5/6, positivity was defined as ≥30% of tumor cells with moderate (++) or strong (+++) membranous/cytoplasmic staining while negative staining was defined as weak-only staining or <30% of tumor cells positive. Using the above criteria, we defined PDAC tumors as having a classical phenotype (GATA6 positive, p40 and CK5/6 negative), basal phenotype (p40 and/or CK5/6 positive with GATA6 negative/low), or a mixed/indeterminant phenotype with co-expression patterns (*e.g*. GATA6 positive with p40 and/or CK5/6 positive) or all three markers negative.

### Next-Generation Sequencing and Analysis

Samples were processed using the FDA-authorized MSK-IMPACT targeted sequencing panel using methods as previously described^23,54^. Briefly, genomic DNA (gDNA) from pathologist-reviewed FFPE tissue and matched normal (peripheral blood) samples were extracted and targeted sequencing was performed with custom DNA probes against all exons and selected introns of a given panel of genes. Most tumors (28/29, 97%) were sequenced using the newest generation of MSK-IMPACT targeting 505 genes, with one tumor sample sequenced on a past generation of MSK-IMPACT targeting 410 genes. Median sequencing depth was 503x (range 184–1100x) per tumor and 474x (220 - 788x) per normal (Table S4). Additionally, for *N* = 1 *KRAS* WT sample, tumor RNA was profiled for actionable fusions using MSK-Fusion which utilizes Archer Anchored Multiplex PCR technology as previously described^55^. Germline variants were classified according to the guidelines from the American College of Medical Genetics and Genomics (ACMG) and the Association for Molecular Pathology (AMP) in 86 cancer susceptible genes (Table S5)^56^.

Tumor purity was estimated by FACETS using the “hisens” mode^57^. If the purity was unevaluable, “purity” mode was considered. If both solutions resulted in unevaluable purity (NA or 0.3), purity was calculated by multiplying the third quartile of the variant allele frequencies of the somatic variants observed in the diploid regions of the sample by 2. Samples with at least 2 mutations were included for this metric. All samples that did not have enough metrics to estimate purity were deemed unevaluable (*N* = 4). For those evaluable (*N* = 25), median tumor purity was estimated at 39% (25^th^ percentile: 26%; 75^th^ percentile: 55%).

Somatic alterations were classified as oncogenic, likely oncogenic, or unknown using OncoKB^58,59^. Copy number calls were evaluated using FACETS tool. All fits were manually reviewed, and the best fits were selected. For paired tumors, variants identified in one of the sample pairs were genotyped in the other sample using GetBaseCounts (https://github.com/zengzheng123/GetBaseCounts). Genes were included in the oncoprint in Fig. 3A if they were mutated in > 1 patient, were the only shared mutation across paired tumors, or are known PDAC driver alterations. PDAC driver mutations were defined as oncogenic alterations in the MAPK pathway (*e.g., KRAS, NRAS, BRAF, FGFR2), TP53, CDKN2A, or SMAD4*.

For the pan-cohort analyses, proportions of oncogenic KRAS, TP53, CDKN2A/B, and SMAD4 alterations were compared to Varghese, Perry et al. (2025)^4^. Publicly available data (https://www.cbioportal.org/study/summary?id=pdac_msk_2024) was curated to include only patients with ‘Stage at Diagnosis’: Resectable, ‘Resection Surgery’: True, ‘Disease Status at Sequencing’: Primary, and ‘Sample Type’: Primary (N = 620 patients). To enhance the detection of sub-threshold *KRAS* mutations and directly match the methods, of Varghese, Perry et al. (2024), only where explicitly discussed in the text, *KRAS* mutations were called if the raw count of ≥ 5 base level *KRAS* variant reads with ≥ 2 of those reads both high-quality and high-confidence. High quality was defined as the read itself being high-quality according the bam file. High-confidence was defined as the base-level *KRAS* variant found in the middle of the read, no other base-level variants found in the read, not a duplicated read, and not all reads were in the same direction.

### Digital Droplet PCR

Assays specific for the detection of G12D, G12R, G12V, and Q61H in KRAS, TP53 E326*, and FBXW7 R505C were ordered through Bio-Rad (Table S6). Cycling conditions were tested with a known positive control to ensure optimal annealing and extension temperature as well as optimal separation of positive from empty droplets. After PicoGreen quantification, 1.5–9 ng gDNA was combined with locus-specific primers, FAM- and HEX-labeled probes, MseI or HaeIII (Q61H only), and digital PCR Supermix for probes (no dUTP). All reactions were performed on a QX200 ddPCR system (Bio-Rad catalog # 1864001) and each sample was evaluated in technical duplicates when mass allowed. Reactions were partitioned into a median of ~22,000 droplets per well using the QX200 droplet generator. Emulsified PCRs were run on a 96-well thermal cycler using cycling conditions identified during the optimization step (95 °C 10 m; 40 cycles of 94 °C 30 s and 55 °C 1 m (KRAS) or 52 C 1 m (TP53, FBXW7); 98 °C 10’; 4 °C hold). Plates were read and analyzed with the QuantaSoft software to assess the number of droplets positive for mutant DNA, wild-type DNA, both, or neither.

### Clinical Relevance

Elucidating the relatedness of dual pancreatic cancers informs clinical decision-making, particularly in this era of genomic biomarker-driven therapies. Independent metachronous pancreatic cancers do rarely occur and support somatic profiling of individual lesions and necessitate identification of actionable variants. Patients with isolated pancreatic metastases are enriched for more favorable disease biology and benefit from systemic and local therapies.

## Supplementary information

Supplementary Information

## Data Availability

Integrated somatic profiling will be made publicly available at https://www.cbioportal.org/study/summary?id=paad_msk_2025 at the time of final publication.
