# Supplementary material for: Dual pancreatic carcinomas: clonally related or independent primaries?
Source: NPJ Precis Oncol. 2026 Feb 8;10:112. doi: 10.1038/s41698-026-01313-4 (PMC12996456; doi:10.1038/s41698-026-01313-4)
Supplement: Supplementary file 1 — Supplementary Information [file 41698_2026_1313_MOESM1_ESM.docx]

**
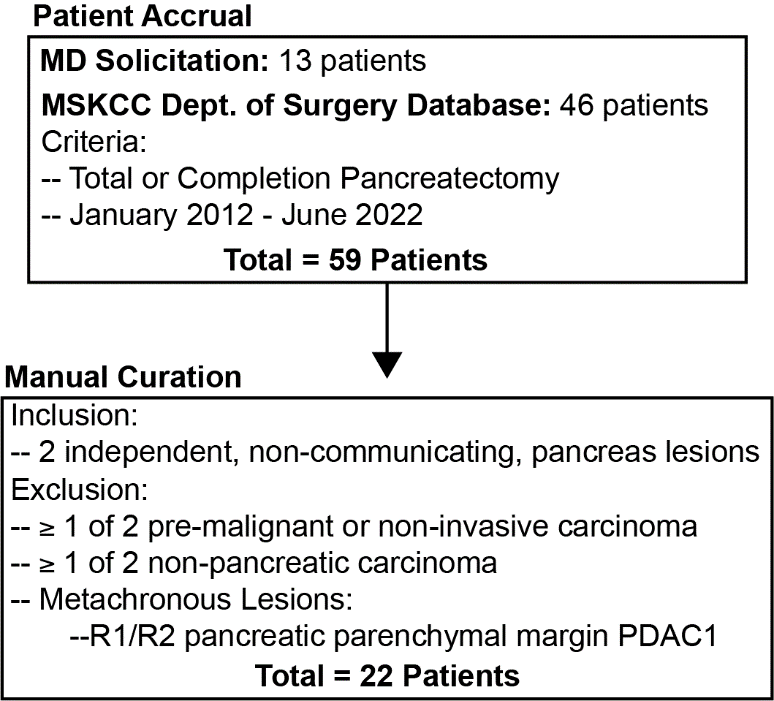
Figure S1. Patient accrual schema.** Patients from Memorial Sloan Kettering Cancer Center (MSK) were accrued retrospectively as approved by the Institutional Review Board. Patients were identified either through direct solicitation from MSK physicians or review of institutional databases. Specifically, the Hepatopancreaticobiliary Surgery Division patient database was reviewed for all patients who had undergone completion or total pancreatectomy since 2012. Participant lists were then manually curated for patients with two invasive carcinomas of the exocrine pancreas; patients with at least one non-invasive lesion or non-exocrine pancreatic carcinoma histology were excluded. For synchronous lesions, participant inclusion required the absence of radiographic or pathologic (if resected) connection between the lesions. For metachronous lesions, patients were excluded if there was confirmed invasive carcinoma or non-invasive carcinoma present at the pancreatic margin at the time of resection of PDAC1; precursor lesions with any degree of dysplasia at the resection margin were allowed.

**
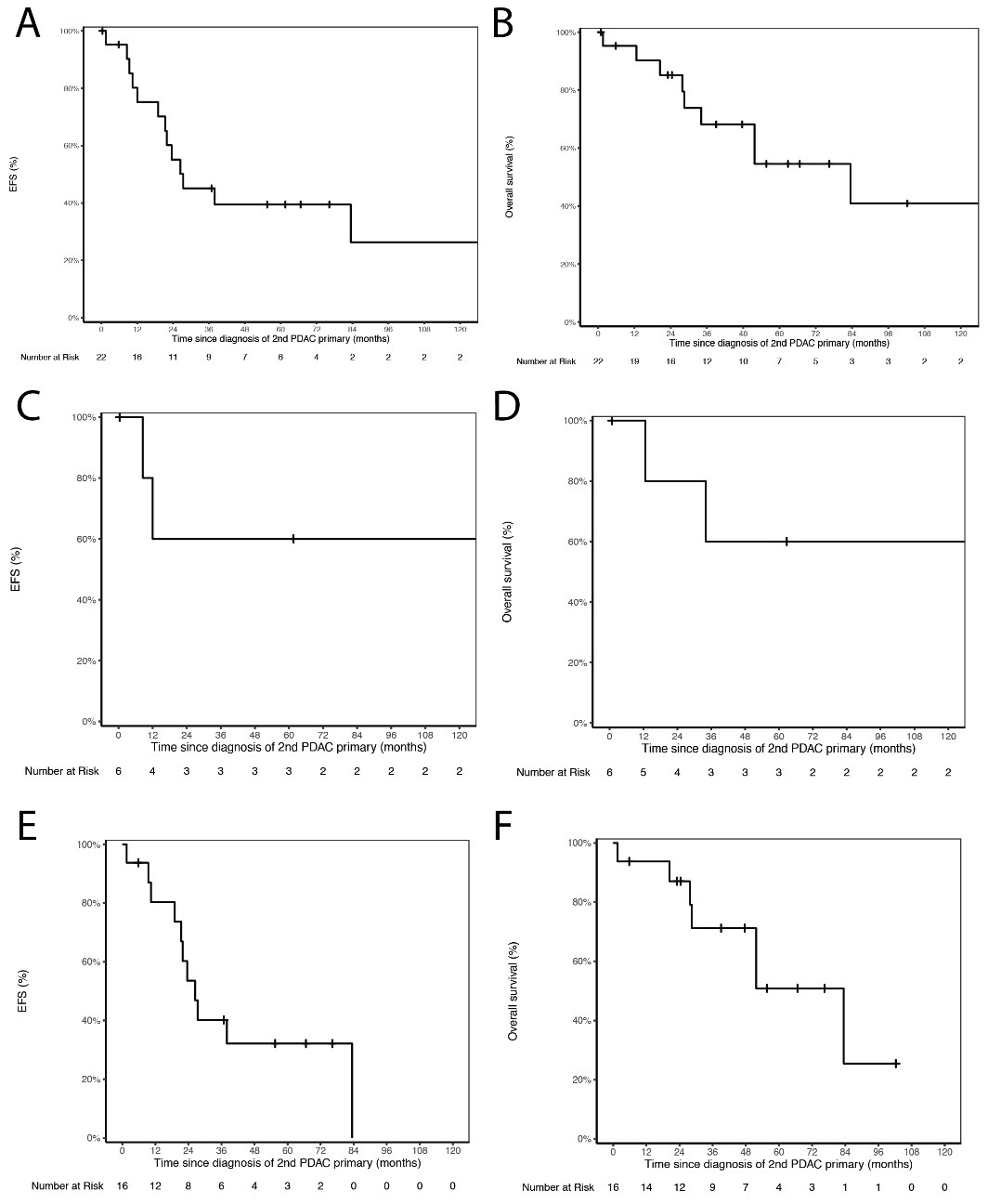
Figure S2. Event free survival (EFS) and Overall Survival (OS) from PDAC2. (A-B)** For the full cohort, at a median follow-up of 55.7 m (range 0.9 – 146.4 m) from PDAC2, 11 patients have recurrent/metastatic disease with 12-, 24-, and 36-month EFS rates of 80%, 55%, and 45% and 12-, 24-, and 36-month OS rates of 95%, 85%, and 68%, respectively. **(C-D)** For patients with synchronous occurring lesions, at a median follow-up of 99.7 m (range 0.9 – 146.4 m), 2 patients have recurrent/metastatic disease with 12-, 24-, and 36-month EFS rates of 80%, 60%, and 60% and 12-, 24-, and 36-month OS rates of 100%, 80%, and 60%, respectively. **(E-F)** For patients with metachronous occurring PDAC, at a median follow-up of 47.6 m (range 5.8 – 102.3 m), 9 patients have recurrent/metastatic disease, with 12-, 24-, and 36-month event free survival (EFS) rates of 80%, 54%, and 40% and 12-, 24-, and 36-month overall survival (OS) rates of 94%, 87%, and 71%, respectively.

**
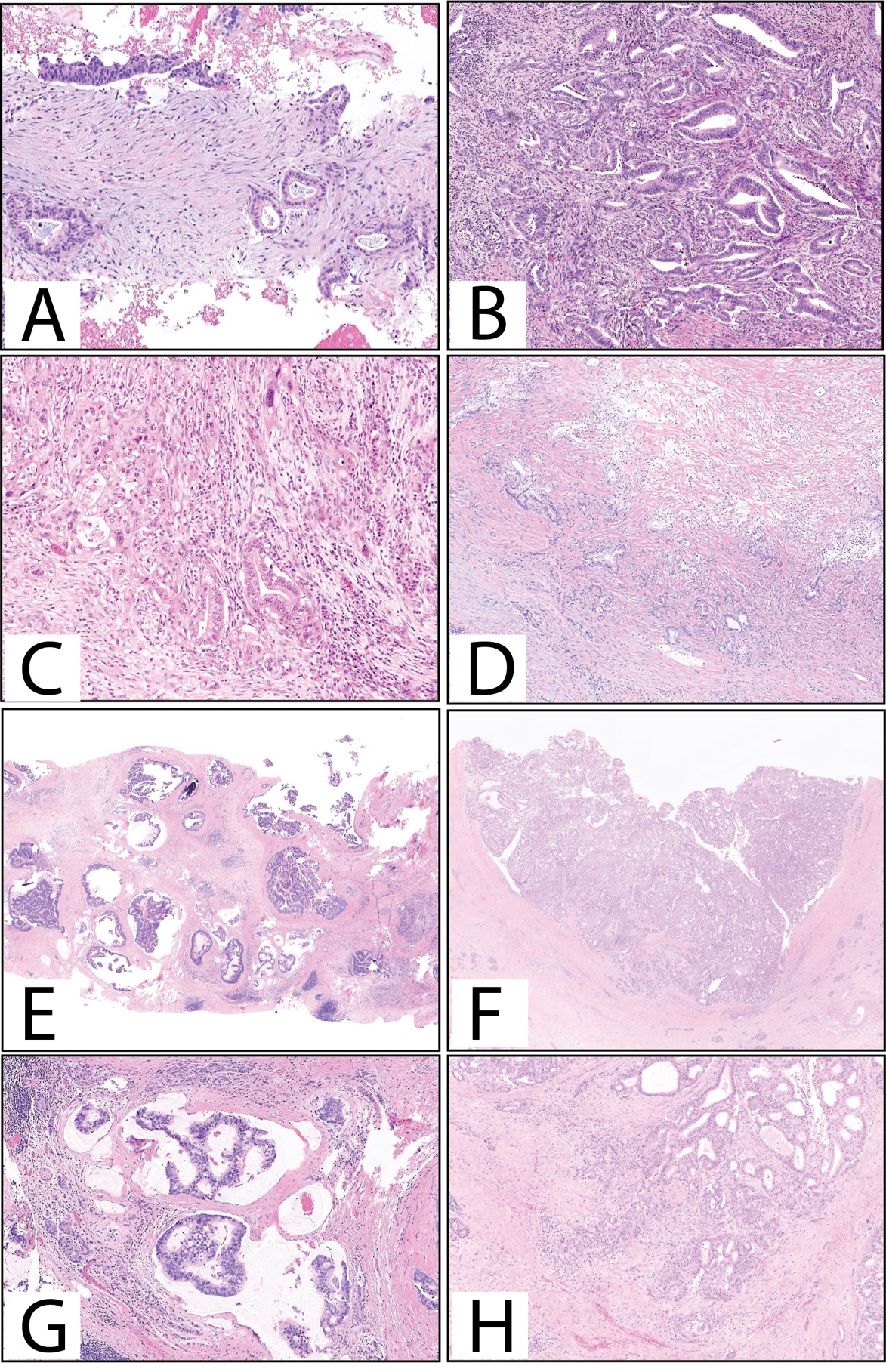
**

**Figure S3. Example H&E of observed histologies.** (**A**-**B**) Pancreatic ductal adenocarcinoma. (A) Patient 7, PDAC1: Pancreatic tail, core biopsy. Small, angulated glands with enlarged, irregular nuclei and attenuated cytoplasm infiltrate a densely desmoplastic stroma. A mild, patchy lymphocytic infiltrate is also present within the stroma. (B) Pancreatic tail, resection specimen**.** Invasive carcinoma forming ill-defined, irregular glands arranged haphazardly within the stroma. Peritumoral stromal activation with abundant fibroblasts and collagen deposition is prominent. A moderate lymphocytic infiltrate is present at the tumor–stroma interface, with neutrophils also observed. (**C**) Pancreatic ductal adenocarcinoma (PDAC) with treatment effect after neoadjuvant therapy. Patient 1: Resection specimen showing residual invasive carcinoma identified as scattered, angulated glands and single atypical cells embedded within therapy-induced fibroinflammatory stroma. Foamy histiocytes and hemosiderin-laden macrophages are interspersed, with diminished viable tumor cellularity compared with untreated PDAC. (**D**) Pancreatic ductal adenocarcinoma (PDAC) with focal squamous differentiation. Patient 21, PDAC2: The resection specimen reveals a biphasic morphology, comprising (i) gland-forming adenocarcinoma with irregular, angulated glands and intracytoplasmic mucin, and (ii) squamous areas composed of solid nests with eosinophilic cytoplasm, keratinization, and distinct intercellular bridges. Nuclear pleomorphism is marked. Lymphocytes and neutrophils are present within the stroma. (**E,F**) Colloid carcinoma arising in association with intraductal papillary mucinous neoplasm (IPMN), intestinal type. Patient 8, PDAC1. (E) Low power (×40) shows lakes of extracellular mucin dissecting pancreatic parenchyma adjacent to cystically dilated ducts lined by papillary mucinous epithelium with intestinal-type morphology, consistent with an associated IPMN. (F) At intermediate power (×100), cohesive clusters and delicate strands of neoplastic epithelial cells are seen “floating” within mucin pools, with focal cribriform fragments. The intervening stroma is relatively scant and only mildly desmoplastic compared with conventional PDAC. A sparse, patchy lymphocytic infiltrate is present at the tumor–stroma interface. (**G,H**) Invasive carcinoma arising in association with intraductal tubulopapillary neoplasm (ITPN). Patient 15. (G) At low power (×40), an intraductal, back-to-back tubular/tubulopapillary proliferation expands native ducts, with sharply circumscribed intraductal nodules and limited/no extracellular mucin, consistent with ITPN. Adjacent invasive carcinoma infiltrates the surrounding parenchyma in irregular, angulated glands embedded within a desmoplastic stroma. (H) At intermediate power (×100), invasive glands demonstrate cribriforming and complex tubules with eosinophilic cytoplasm.

**
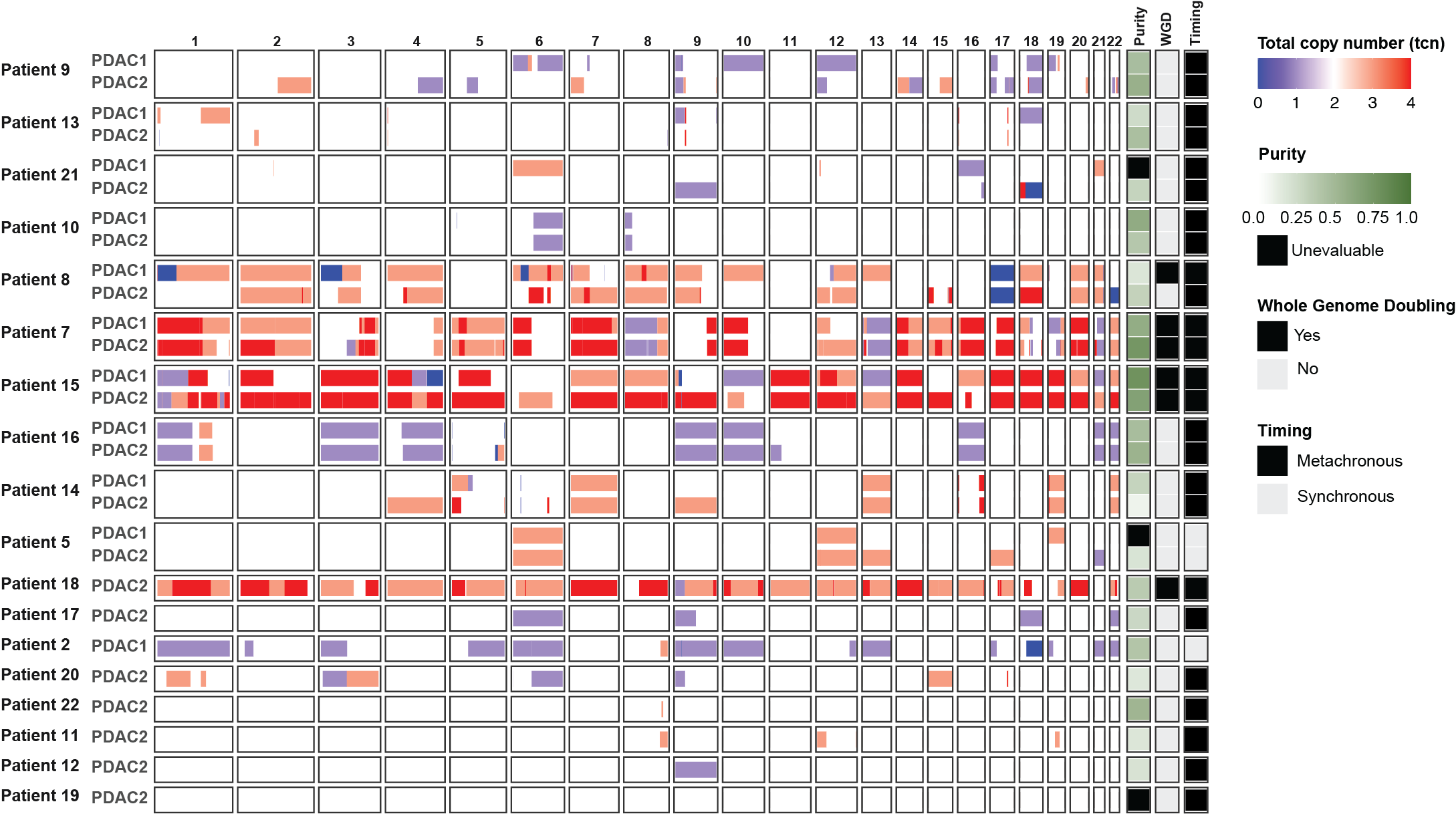
Figure S4. Copy number alteration profiles of synchronous and metachronous occurring PDAC.**

**
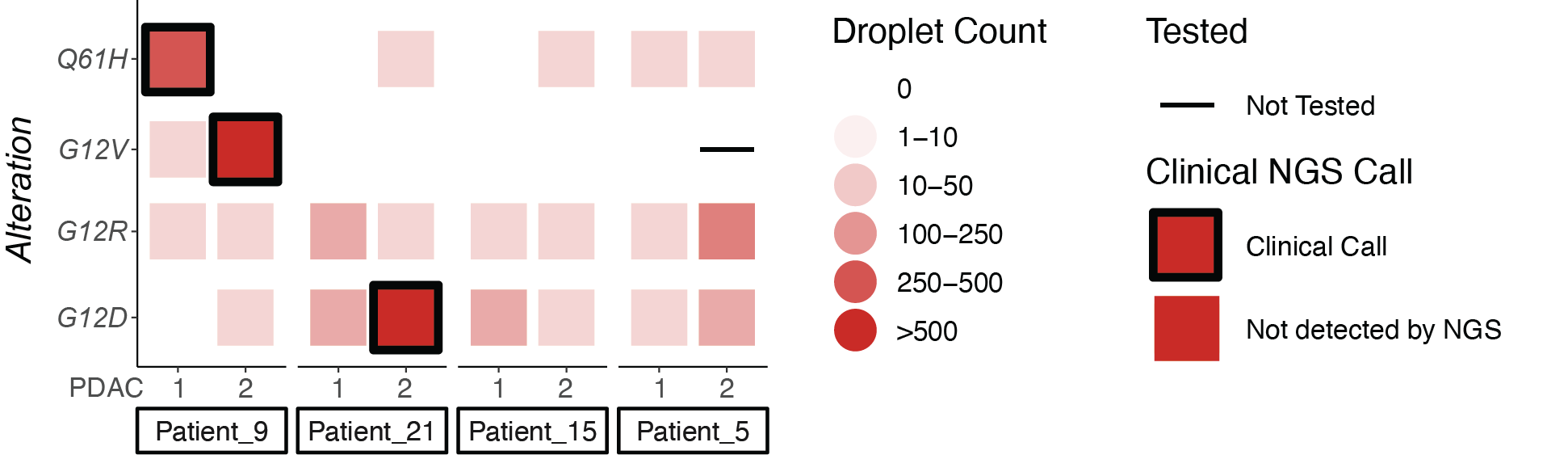
Figure S5. *KRAS* hotspot ddPCR identifies multiple low VAF oncogenic mutations in all samples tested.** Extracted bulk gDNA was assayed for *KRAS* G12D, G12V, G12R, and Q61H mutations by ddPCR for all paired samples for which an oncogenic driver mutation was not identified by NGS. Additionally, clonally independent tumors from patient 9 were assayed to evaluate for low VAF shared mutations.

**Table S1. Histopathologic summary of synchronous and metachronous dual pancreas cancers**

| **ID** | **Timing (m)** | **PDAC** | **Location** | **Histopathology** | **Differentiation** | **Precursor Lesion (Subtype)** | **T**  **Stage** | **N Stage** | **TNM**  **Stage**  **Stage** | **Margin Status** | **Tx**  **Effect** | **Subtyping IHC** | | | |
| --- | --- | --- | --- | --- | --- | --- | --- | --- | --- | --- | --- | --- | --- | --- | --- |
|  |  |  |  |  |  |  |  |  |  |  |  | **p40** | **GATA6** | **CK5/6** | **Subtype** |
| **1** | Synch. | 1 | Head | Post-treatment residual PDAC | NA | IPMN (pancreaticobiliary) | ypT1b | ypN1 | IIB | Negative | 2 |  |  |  |  |
|  |  | 2 | Tail | Post-treatment residual PDAC | Moderate | IPMN (pancreaticobiliary) | ypT2 |  |  | Negative | 3 |  |  |  |  |
| **2** | Synch. | 1 | Head | PDAC | NA | NR | NR | NR | NR | NR |  |  |  |  |  |
|  |  | 2 | Tail | PDAC | Poor | NR | NR |  |  | NR |  |  |  |  |  |
| **3** | Synch. | 1 | Head | PDAC | Moderate | NA | ypT3 | ypN0 | IIA | NA |  |  |  |  |  |
|  |  | 2 | Tail | PDAC | Moderate | NA | ypT1a |  |  | NA |  |  |  |  |  |
| **4** | Synch. | 1 | Head | Post-treatment residual PDAC | Moderate | PanIN | ypT2 | ypN1 | IIB | Negative | 2 | Negative | +++ (100%) | ++ (1%) | Classical |
|  |  | 2 | Tail | PDAC | NA | NA | pCR |  |  | Negative | 0 |  |  |  |  |
| **5** | Synch. | 1 | Head | Post-treatment residual PDAC | Well | PanIN | ypT2 | ypN1 | IIB | Negative (LG PanIN, distal pancreatic) | 2 | Negative | +++ (100%) | Negative | Classical |
|  |  | 2 | Tail | Post-treatment residual PDAC | Well | PanIN | ypT1c |  |  | Negative (LG PanIN, distal pancreatic) | 2 | Negative | +++ (100%) | + (30%) | Classical |
| **6** | Synch. | 1 | Head | PDAC | Poor | PanIN | pT1c | pN0 | IB | Negative (LG PanIN, distal pancreatic) |  |  |  |  |  |
|  |  | 2 | Tail | PDAC | Poor | PanIN | pT2 |  |  | Negative |  |  |  |  |  |
| **7** | Metach. | 1 | Tail | PDAC | Moderate | PanIN | pT1c | pN0 | IA | Negative |  |  |  |  |  |
|  | 17 m | 2 | Head | Colloid Carcinoma | Moderate | IPMN (intestinal) | pT1b | pN0 | IA | Negative |  | Negative | ++ (100%) | + (1%) | Classical |
| **8** | Metach. | 1 | Tail | Colloid Carcinoma | Moderate | IPMN (intestinal) | pT1b | pN0 | IA | Negative (LG PanIN, proximal margin) |  | Negative | +++ (100%) | Negative | Classical |
|  | 33 m | 2 | Head | PDAC | Moderate | PanIN | pT3 | pN2 | III | Negative |  | Negative | ++ (100%) | Negative | Classical |
| **9** | Metach. | 1 | Head | PDAC, focal squamous features | Poor | PanIN | pT2 | pN1 | IIB | Negative |  | Negative | ++ (60%) | ++ (80%) | Mixed |
|  | 14 m | 2 | Tail | Post-treatment residual PDAC | Moderate | PanIN | ypT2 | ypN1 | IIB | Negative | 3 | Negative | ++ (90%) | ++ (60%) | Mixed |
| **10** | Metach. | 1 | Head | Colloid Carcinoma | Moderate | IPMN (intestinal) | pT2 | pN0 | IB | Negative |  | Negative | +++ (100%) | Negative | Classical |
|  | 26 m | 2 | Tail | Colloid Carcinoma | Moderate | IPMN (intestinal) | pT2 | pN0 | IB | Negative |  | Negative | +++ (100%) | Negative | Classical |
| **11** | Metach. | 1 | Head | PDAC | NA | NA | pCR | ypN0 |  | Negative | 0 |  |  |  |  |
|  | 34 m | 2 | Tail | PDAC | Moderate | PanIN | pT2 | pN0 | IB | Negative |  | Negative | ++ (80%) | ++ (5%) | Classical |
| **12** | Metach. | 1 | Head | Post-treatment residual PDAC | Moderate | PanIN | ypT2 | ypN0 | IB | Negative | 2 |  |  |  |  |
|  | 18 m | 2 | Tail | PDAC | Poor | PanIN | pT2 | pN0 | IB | Negative |  |  |  |  |  |
| **13** | Metach. | 1 | Tail | Post-treatment residual PDAC | Moderate | PanIN | ypT3 | ypN0 | IIA | Negative | 2 | Negative | ++ (60%) | Negative | Classical |
|  | 22 m | 2 | Head | PDAC, large duct type | Moderate | PanIN | pT2 | pN0 | IB | Negative |  |  |  |  |  |
| **14** | Metach. | 1 | Head | PDAC | NA | PanIN | pT1a | pN0 | IA | Negative (HG PanIN, distal pancreatic) |  | Negative | +++ (100%) | Negative | Classical |
|  | 16 m | 2 | Tail | PDAC | Moderate | IPMN (intestinal) | pT2 | pN0 | IB | Positive, Posterior-inferior pancreatic |  | Negative | ++/+++ (100%) | Negative | Classical |
| **15** | Metach. | 1 | Head | PDAC | Moderate | ITPN | pT1 | pN0 | IA | Negative |  | ++ (5%) | +++ (95%) | + (5%) | Classical |
|  | 62 m | 2 | Tail | PDAC | NA | ITPN | pT1b | pN0 | IA | Negative |  | ++ (1%) | +/++ (80%) | Negative | Classical |
| **16** | Metach. | 1 | Head | PDAC | Moderate | ITPN | pT1 | pN0 | IA | Negative |  | Negative | +++ (100%) | Negative | Classical |
|  | 30 m | 2 | Tail | PDAC | Moderate | ITPN | pT1 | pN0 | IA | Negative |  | Negative | +++ (100%) | Negative | Classical |
| **17** | Metach. | 1 | Head | PDAC | Moderate | PanIN | pT2 | pN0 | IB | Negative |  |  |  |  |  |
|  | 87 m | 2 | Tail | PDAC | Poor | PanIN | pT2 | pN1 | IIB | Negative |  |  |  |  |  |
| **18** | Metach. | 1 | Head | Post-treatment residual PDAC | Moderate | IPMN | ypT2 | ypN0 | IB | Negative | 2 | Negative | +++ (100%) | Negative | Classical |
|  | 101 m | 2 | Tail | PDAC | Moderate/Poor | Suggestive of IPMN | pT2 | pN1 | IIB | Negative |  | Negative | +++ (60%) | ++ (2-3%) | Classical |
| **19** | Metach. | 1 | Tail | PDAC | NA | NA | pT1 | pN0 | IA | NA |  |  |  |  |  |
|  | 69 m | 2 | Head | PDAC | Moderate | PanIN | pT2 | pN1 | IIB | Negative |  |  |  |  |  |
| **20** | Metach. | 1 | Tail | PDAC, with cystic degeneration | Moderate | Can’t exclude IPMN, Denuded | pT1 | pN0 | IA | Positive, Posterior/deep (soft tissue) |  | Negative | +++ (80%) | Negative | Classical |
|  | 99 m | 2 | Head | PDAC | Moderate/Poor | PanIN | pT1c | pN0 | IA | Positive, Uncinate |  |  |  |  |  |
| **21** | Metach. | 1 | Tail | PDAC | Moderate/Poor | PanIN | pT2 | pN0 | IB | Negative |  |  |  |  |  |
|  | 63 m | 2 | Head | PDAC, focal squamous features | Moderate/Poor | PanIN | pT2 | pN1 | IIB | Negative |  |  |  |  |  |
| **22** | Metach. | 1 | Head | PDAC | NA | NA | pT3 | pN1 | IIB | NA |  |  |  |  |  |
|  | 151 m | 2 | Tail | PDAC | Moderate | PanIN | pT1c | pN1 | IIB | Negative |  | Negative | +++ (100%) | Negative | Classical |
| Synch.: Synchronous; Metach.: Metachronous; Resect.: Resection; FNA: Fine Needle Aspirate; FNB: Fine Needle Biopsy; PDAC: Pancreatic Ductal Adenocarcinoma; NR: Not Resected; NA: Not Available/Applicable | | | | | | | | | | | | | | | |
| IPMN: Intraductal Papillary Mucinous Neoplasm^59^; IAPN: Intraampullary Papillary Neoplasm^60^; ITPN: Intraductal Tubulopapillary Neoplasm^61, 62^; PanIN: Pancreatic Intraepithelial Neoplasia; LG: Low Grade; HG: High Grade | | | | | | | | | | | | | | | |
| Treatment (Tx) Effect^63^: 0: Complete Response, 1: Near complete response , 2: Partial response, 3: No/poor response. | | | | | | | | | | | | | | | |
| IHC Staining Intensity (% Positive Cells): + (mild), ++ (moderate), +++ (strong) | | | | | | | | | | | | | | | |

| **Table S2. Summary ddPCR Results: Mutant and WT droplet counts.** | | | | | | | | | | | | | | | | | | | |
| --- | --- | --- | --- | --- | --- | --- | --- | --- | --- | --- | --- | --- | --- | --- | --- | --- | --- | --- | --- |
|  |  | **KRAS Q61H** | | | **KRAS G12V** | | | **KRAS G12D** | | | **KRAS G12R** | | | **TP53 E326*** | | | **FBXW7 R505C** | | |
| **Patient** | **Tumor** | **# Mut** | **# WT** | **VAF** | **# Mut** | **# WT** | **VAF** | **# Mut** | **# WT** | **VAF** | **# Mut** | **# WT** | **VAF** | **# Mut** | **# WT** | **VAF** | **# Mut** | **# WT** | **VAF** |
| 2 | PDAC2 | 0 | 978 | 0.00% | 0 | 702 | 0.00% | 230 | 712 | 24.42% | 2 | 753 | 0.26% |  |  |  |  |  |  |
| 5 | PDAC1 | 1 | 3970 | 0.03% | 0 | 315 | 0.00% | 5 | 343 | 1.44% | 1 | 371 | 0.27% |  |  |  |  |  |  |
|  | PDAC2 | 1 | 9213 | 0.01% |  |  |  | 28 | 7886 | 0.35% | 134 | 7376 | 1.78% |  |  |  |  |  |  |
| 9 | PDAC1 | 455 | 1163 | 28.12% | 1 | 2471 | 0.04% | 0 | 2449 | 0.00% | 1 | 2418 | 0.04% |  |  |  |  |  |  |
|  | PDAC2 | 0 | 3477 | 0.00% | 1335 | 2581 | 34.09% | 2 | 2614 | 0.08% | 10 | 2706 | 0.37% |  |  |  |  |  |  |
| 15 | PDAC1 | 0 | 4535 | 0.00% | 0 | 4584 | 0.00% | 18 | 4640 | 0.39% | 5 | 4234 | 0.12% |  |  |  |  |  |  |
|  | PDAC2 | 2 | 2928 | 0.07% | 0 | 1250 | 0.00% | 3 | 1339 | 0.22% | 1 | 1318 | 0.08% |  |  |  |  |  |  |
| 21 | PDAC1 | 0 | 2425 | 0.00% | 0 | 2489 | 0.00% | 11 | 2488 | 0.44% | 41 | 2456 | 1.64% | 52 | 7140 | 0.72% | 39 | 8320 | 0.47% |
|  | PDAC2 | 4 | 2875 | 0.14% | 0 | 3203 | 0.00% | 502 | 3451 | 12.70% | 3 | 3365 | 0.09% |  |  |  |  |  |  |

| **Table S3. Summary of the literature regarding genetics of synchronous and metachronous occurring PDAC.** | | | | | | | | | | | | |
| --- | --- | --- | --- | --- | --- | --- | --- | --- | --- | --- | --- | --- |
|  |  |  | **PDAC #1** | | | | **PDAC #2** | | | |  |  |
| **Study** | **Germline PV** | **Timing. (Interval, m)** | **Histology** | **Location** | **Genetics** | | **Histology** | **Location** | **Genetics** | | **Study Adjudication** | **Re-Adjudication** |
| Luchini et al. (2018) | Unknown | Metachronous (50) | Conventional | Head | *KRAS* G12V | | Undiff. with osteoclasts | Body | *KRAS* G12D, *TP53* R290Pfs | | Independent | Likely Independent |
|  | Unknown | Metachronous (36) | Conventional | Body | *KRAS* G12R | | Conventional | Head | *KRAS* G12R | | Related | Likely Related |
|  | Unknown | Metachronous (48) | Cribriform | Head | *KRAS* G12V | | Conventional | Tail | *KRAS* G12D | | Independent | Likely Independent |
|  | Unknown | Metachronous (36) | Conventional | Head | *KRAS* G12D | | Micropapillary | Body | *KRAS* G12D | | Undetermined | Likely Related |
| Iwakura et al (2018) | Unknown | Metachronous (23) | Conventional | Head | *KRAS* G12V | | Conventional | Tail | *KRAS* G12V | | Related | Likely Related |
|  | Unknown | Metachronous (48) | Conventional | Body | *KRAS* G12D | | Conventional | Head | *KRAS* G12D | | Independent | Likely Related |
| Bauer et al. (2018) | Unknown | Metachronous (72) | Conventional | Head | *KRAS* G12R, *TP53* Y220C, *CDKN2A* splice c.151-1G>C, *ACVR1B* | | Conventional | Tail | *KRAS* G12R, *TP53* Y220C, *CDKN2A* splice c.151-1G>C, *myc* amplification | | Undetermined | Related |
| Connor et al. (2019)^a^ | Negative | Metachronous (15) | Conventional | Head | *KRAS* G12R, *TP53* V140M and V272M, *SMAD4* loss, *CDKN2A* loss | | Conventional (Two tumors) | Body, Tail | *KRAS* G12R, *TP53* V140M and V272M, *SMAD4* loss, *CDKN2A* loss | | Related | Related |
|  | Unknown | Metachronous | Conventional | Head | *KRAS* G12R, *SMAD4* R34*/R349*/R445*, *PTEN* H185D | | Conventional | Tail | *KRAS* G12R, *SMAD4* R34*/R349*/R445*, *PTEN* H185D | | Related | Related |
|  | Unknown | Metachronous | Conventional | Head | *KRAS* G12R, *TP53* R282W/R123W/R243W, *KDM6A* (multiple mutations) | | Conventional | Tail | *KRAS* G12R, *TP53* R282W/R123W/R243W, *SMAD4* Y95H | | Related | Related |
|  | Unknown | Metachronous | Conventional | Head | *KRAS* G12D, *TP53* M237I/M78I/M198I/M144I/M105I | | Conventional | Tail | *KRAS* G12D, *TP53* M237I/M78I/M198I/M144I/M105I | | Related | Related |
|  | gPALB2, with LOH | Synchronous | Conventional | Head | *KRAS* G12V, *PALB2* Q430* (somatic LOH) | | Conventional | Unknown | *KRAS* G12V, *PALB2* Q430* (somatic LOH) | | Related | Related |
|  | Unknown | Synchronous | Conventional | Unknown | *KRAS* G12V,  *TP53* E204*/E45*/E165*/E72*,  *SMAD4* S178*/S23* | | Conventional | Unknown | *KRAS* G12V,  *TP53* E204*/E45*/E165*/E72*,  *SMAD4* S178*/S23*, *CDKN2A* R131* | | Related | Related |
|  | Unknown | Synchronous | Conventional | Head | *KRAS* Q61H, *TP53* R183*/R303*/R342*, *TET1* K1524Q | | Conventional | Tail | *KRAS* Q61H, *TP53* R183*/R303*/R342*, *TET1* K1524Q | | Related | Related |
| Fujita et al.  (2020) | Unknown | Synchronous | Conventional | Tail | *KRAS G1R, TP53 E294fs* | | Conventional | Body | *KRAS G12D, TP53 F113C* | | Independent | Independent |
|  | Unknown | Synchronous | Conventional | Tail | *KRAS G12D, FLT S985F* | | Conventional | Body | *KRAS G12V, SMAD4 R135*,*  *TP53 R196** | | Independent | Independent |
|  | Unknown^a^ | Synchronous and Metachronous (43) | Conventional  (Synch.)  Conventional | Tail  Body | *KRAS Q61H, ATM A1733T, MLH1 A380T, SMAD4 G393D/G508D/Q388*, TP53 G293E/Q100**  *ATM D2997N, V3020M, SMAD4 P346H, TP53 E298K/G356R/P222S/P4S/R337H/S6L* | | Conventional  (Metach.) | Head | *KRAS Q61H/R149K/V9I, SMAD4 C127R/S191L, TP53 E294** | | Independent | Independent |
|  | Unknown | Synchronous | Conventional | Tail | *CDKN2A E120*, KDR Q119K, PTEN Q245*, TP53 T205C, JAK2 R922G* | |  | Body | *CDKN2A E120*, KDR Q119K, PTEN Q245*, TP53 T205C* | | Related | Related |
| ^a^Only putative driver mutations presented. | | | | |  |  |  | |  |  |  |  |

| **Table S4. IMPACT 505 Target Genes** | | | | | | | | | |
| --- | --- | --- | --- | --- | --- | --- | --- | --- | --- |
| ABL1 | CALR | DICER1 | FGFR1 | HLA-C | MALT1 | NOTCH1 | PPP4R2 | RXRA | SUZ12 |
| ACVR1 | CARD11 | DIS3 | FGFR2 | HNF1A | MAP2K1 | NOTCH2 | PPP6C | RYBP | SYK |
| AGO1 | CARM1 | DNAJB1 | FGFR3 | HOXB13 | MAP2K2 | NOTCH3 | PRDM1 | SCG5 | TAP1 |
| AGO2 | CASP8 | DNMT1 | FGFR4 | HRAS | MAP2K4 | NOTCH4 | PRDM14 | SDHA | TAP2 |
| AKT1 | CBFB | DNMT3A | FH | ICOSLG | MAP3K1 | NPM1 | PREX2 | SDHAF2 | TBX3 |
| AKT2 | CBL | DNMT3B | FLCN | ID3 | MAP3K13 | NRAS | PRKAR1A | SDHB | TCEB1 |
| AKT3 | CCND1 | DOT1L | FLT1 | IDH1 | MAP3K14 | NSD1 | PRKCI | SDHC | TCF3 |
| ALB | CCND2 | DROSHA | FLT3 | IDH2 | MAPK1 | NTHL1 | PRKD1 | SDHD | TCF7L2 |
| ALK | CCND3 | DUSP4 | FLT4 | IFNGR1 | MAPK3 | NTRK1 | PTCH1 | SERPINB3 | TEK |
| ALOX12B | CCNE1 | E2F3 | FOXA1 | IGF1 | MAPKAP1 | NTRK2 | PTEN | SERPINB4 | TERT |
| ANKRD11 | CD274 | EED | FOXF1 | IGF1R | MAX | NTRK3 | PTP4A1 | SESN1 | TET1 |
| APC | CD276 | EGFL7 | FOXL2 | IGF2 | MCL1 | NUF2 | PTPN11 | SESN2 | TET2 |
| APLNR | CD79A | EGFR | FOXO1 | IKBKE | MDC1 | NUP93 | PTPRD | SESN3 | TGFBR1 |
| AR | CD79B | EIF1AX | FOXP1 | IKZF1 | MDM2 | PAK1 | PTPRS | SETD2 | TGFBR2 |
| ARAF | CDC42 | EIF4A2 | FUBP1 | IL10 | MDM4 | PAK7 | PTPRT | SETDB1 | TMEM127 |
| ARHGAP35 | CDC73 | EIF4E | FYN | IL7R | MED12 | PALB2 | RAB35 | SF3B1 | TMPRSS2 |
| ARID1A | CDH1 | ELF3 | GAB1 | INHA | MEF2B | PARK2 | RAC1 | SH2B3 | TNFAIP3 |
| ARID1B | CDK12 | EP300 | GAB2 | INHBA | MEN1 | PARP1 | RAC2 | SH2D1A | TNFRSF14 |
| ARID2 | CDK4 | EPAS1 | GATA1 | INPP4A | MET | PAX5 | RAD21 | SHOC2 | TOP1 |
| ARID5B | CDK6 | EPCAM | GATA2 | INPP4B | MGA | PBRM1 | RAD50 | SHQ1 | TP53 |
| ASXL1 | CDK8 | EPHA3 | GATA3 | INPPL1 | MITF | PDCD1 | RAD51 | SLFN11 | TP53BP1 |
| ASXL2 | CDKN1A | EPHA5 | GLI1 | INSR | MLH1 | PDCD1LG2 | RAD51C | SLX4 | TP63 |
| ATM | CDKN1B | EPHA7 | GNA11 | IRF4 | MLLT1 | PDGFRA | RAD51L1 | SMAD2 | TRAF2 |
| ATR | CDKN2A | EPHB1 | GNAQ | IRS1 | MPL | PDGFRB | RAD51L3 | SMAD3 | TRAF7 |
| ATRX | CDKN2B | ERBB2 | GNAS | IRS2 | MRE11A | PDPK1 | RAD52 | SMAD4 | TRIP13 |
| ATXN7 | CDKN2C | ERBB3 | GNB1 | JAK1 | MSH2 | PGBD5 | RAD54L | SMARCA2 | TSC1 |
| AURKA | CEBPA | ERBB4 | GPS2 | JAK2 | MSH3 | PGR | RAF1 | SMARCA4 | TSC2 |
| AURKB | CENPA | ERCC2 | GREM1 | JAK3 | MSH6 | PHF6 | RARA | SMARCB1 | TSHR |
| AXIN1 | CHEK1 | ERCC3 | GRIN2A | JUN | MSI1 | PHOX2B | RASA1 | SMARCD1 | U2AF1 |
| AXIN2 | CHEK2 | ERCC4 | GSK3B | KBTBD4 | MSI2 | PIK3C2G | RB1 | SMARCE1 | UPF1 |
| AXL | CIC | ERCC5 | H3F3A | KDM5A | MST1 | PIK3C3 | RBM10 | SMO | USP8 |
| B2M | CMTR2 | ERF | H3F3B | KDM5C | MST1R | PIK3CA | RECQL | SMYD3 | VEGFA |
| BABAM1 | CREBBP | ERG | H3F3C | KDM6A | MTAP | PIK3CB | RECQL4 | SOCS1 | VHL |
| BAP1 | CRKL | ERRFI1 | HGF | KDR | MTOR | PIK3CD | REL | SOS1 | VTCN1 |
| BARD1 | CRLF2 | ESR1 | HIST1H1C | KEAP1 | MUTYH | PIK3CG | REST | SOX17 | WHSC1 |
| BBC3 | CSDE1 | ETAA1 | HIST1H2BD | KIT | MYC | PIK3R1 | RET | SOX2 | WHSC1L1 |
| BCL10 | CSF1R | ETV1 | HIST1H3A | KLF4 | MYCL1 | PIK3R2 | RFWD2 | SOX9 | WT1 |
| BCL2 | CSF3R | ETV6 | HIST1H3B | KLF5 | MYCN | PIK3R3 | RHEB | SPEN | WWTR1 |
| BCL2L1 | CTCF | EZH1 | HIST1H3C | KMT2A | MYD88 | PIM1 | RHOA | SPOP | XIAP |
| BCL2L11 | CTLA4 | EZH2 | HIST1H3D | KMT2B | MYOD1 | PLCG2 | RICTOR | SPRED1 | XPO1 |
| BCL6 | CTNNB1 | FAM123B | HIST1H3E | KMT2C | NADK | PLK2 | RIT1 | SPRTN | XRCC2 |
| BCOR | CTR9 | FAM175A | HIST1H3F | KMT2D | NBN | PMAIP1 | RNF43 | SRC | YAP1 |
| BIRC3 | CUL3 | FAM46C | HIST1H3G | KMT5A | NCOA3 | PMS1 | ROS1 | SRSF2 | YES1 |
| BLM | CXCR4 | FAM58A | HIST1H3H | KNSTRN | NCOR1 | PMS2 | RPS6KA4 | STAG2 | ZFHX3 |
| BMPR1A | CXORF67 | FANCA | HIST1H3I | KRAS | NEGR1 | PNRC1 | RPS6KB2 | STAT3 | ZNRF3 |
| BRAF | CYLD | FANCC | HIST1H3J | LATS1 | NF1 | POLD1 | RPTOR | STAT5A | ZRSR2 |
| BRCA1 | CYP19A1 | FAT1 | HIST2H3C | LATS2 | NF2 | POLE | RRAGC | STAT5B |  |
| BRCA2 | CYSLTR2 | FBXW7 | HIST2H3D | LMO1 | NFE2L2 | POT1 | RRAS | STK11 |  |
| BRD4 | DAXX | FGF19 | HIST3H3 | LYN | NFKBIA | PPARG | RRAS2 | STK19 |  |
| BRIP1 | DCUN1D1 | FGF3 | HLA-A | LZTR1 | NKX2-1 | PPM1D | RTEL1 | STK40 |  |
| BTK | DDR2 | FGF4 | HLA-B | MAD2L2 | NKX3-1 | PPP2R1A | RUNX1 | SUFU |  |

| **Table S5. Expanded Germline Profiling Gene List** | | | |
| --- | --- | --- | --- |
| *ALK* | *ERBB2* | *NF2* | *SDHC* |
| *APC* | *ERCC3* | *NTHL1* | *SDHD* |
| *ATM* | *ETV6* | *POLD1* | *SDHAF2* |
| *BAP1* | *FAM175A* | *POLE* | *TGFBR1* |
| *BARD1* | *FANCA* | *PALB2* | *TGFBR2* |
| *BMPR1A* | *FANCC* | *PAX5* | *SMAD3* |
| *SMAD4* | *FH* | *PHOX2B* | *TGFBR2* |
| *BRCA1* | *FLCN* | *PTCH1* | *SMARCA4* |
| *BRCA2* | *GATA2* | *PTEN* | *SMARCB1* |
| *BRIP1* | *HOXB13* | *RAD50* | *STK11* |
| *CDH1* | *HRAS* | *RAD51* | *SUFU* |
| *CDC73* | *KRAS* | *RAD51B* | *TERT* |
| *CDKN2A(P16)* | *NRAS* | *RAD51C* | *TMEM127* |
| CDK4 | *KIT* | RAD51D | *TP53* |
| *CEBPA* | *PDGFRA* | *RB1* | *RAD51B* |
| *CHEK2* | *MEN1* | *RECQL* | *RAD51C* |
| *DICER1* | *MET* | *RECQL4* | *RAD51D* |
| *EGFR* | *MITF* | *RET* | *TSC1* |
| *EPCAM* | *MRE11A* | *RTEL1* | *TSC2* |
| *MLH1* | *MUTYH* | *RUNX1* | *VHL* |
| *MSH2* | *NBN* | *SDHA* | *WT1* |
| *MSH6* | *NF1* | *SDHB* | *YAP1* |

| **Table S6. ddPCR Assay Reagents** | |
| --- | --- |
| Assay Name | Assay ID |
| *KRAS* G12D | dHsaCP2000001/2 |
| *KRAS* G12R | dHsaMDS615795490 |
| *KRAS* G12V | dHsaCP2000005 |
| *KRAS* Q61H | dHsaMDS248921642 |
| *TP53* E326* | dHsaMDS2512864 |
| *FBXW7* R505C | dHsaMDV2516774 |

**Supplemental Works Cited**

59. Basturk O, Esposito I, Fukushima N, Furukawa T, Hong SM, Kloppel G, *et al*. WHO Classification of Tumours of the Digestive System: IARC; 2019.

60. Ohike N, Kim GE, Tajiri T, Krasinskas A, Basturk O, Coban I, *et al*. Intra-ampullary papillary-tubular neoplasm (IAPN): characterization of tumoral intraepithelial neoplasia occurring within the ampulla: a clinicopathologic analysis of 82 cases. Am J Surg Pathol. 2010;34(12):1731-48. doi: 10.1097/PAS.0b013e3181f8ff05. PubMed PMID: 21084962; PMCID: PMC3168573.

61. Basturk O, Adsay V, Askan G, Dhall D, Zamboni G, Shimizu M, *et al*. Intraductal Tubulopapillary Neoplasm of the Pancreas: A Clinicopathologic and Immunohistochemical Analysis of 33 Cases. Am J Surg Pathol. 2017;41(3):313-25. doi: 10.1097/pas.0000000000000782. PubMed PMID: 27984235; PMCID: PMC5309137.

62. Basturk O, Berger MF, Yamaguchi H, Adsay V, Askan G, Bhanot UK, *et al*. Pancreatic intraductal tubulopapillary neoplasm is genetically distinct from intraductal papillary mucinous neoplasm and ductal adenocarcinoma. Mod Pathol. 2017;30(12):1760-72. Epub 20170804. doi: 10.1038/modpathol.2017.60. PubMed PMID: 28776573.

63. Burgart LJ, Chopp WV, Jain D. Protocol for the examination of specimens from patients with carcinoma of the pancreas, version 4.2.0.2. Coll Am Pathol. 2021.
